# Environmental and Microbial Interactions Shape Methane-Oxidizing Bacterial Communities in a Stratified Lake

**DOI:** 10.3389/fmicb.2020.579427

**Published:** 2020-10-15

**Authors:** Carole Guggenheim, Remo Freimann, Magdalena J. Mayr, Karin Beck, Bernhard Wehrli, Helmut Bürgmann

**Affiliations:** ^1^ Department of Environmental Systems Science, Institute of Biogeochemistry and Pollutant Dynamics, ETH Zurich - Swiss Federal Institute of Technology, Zurich, Switzerland; ^2^ Department of Surface Waters ‐ Research and Management, Eawag ‐ Swiss Federal Institute of Aquatic Science and Technology, Kastanienbaum, Switzerland; ^3^ Department of Biology, Institute of Molecular Health Sciences, ETH Zurich - Swiss Federal Institute of Technology, Zurich, Switzerland

**Keywords:** methanotrophs, methane oxidation, *pmoA*, bacterial interactions, environmental factors, diversity, habitat specificity

## Abstract

In stratified lakes, methane-oxidizing bacteria (MOB) are strongly mitigating methane fluxes to the atmosphere by consuming methane entering the water column from the sediments. MOB communities in lakes are diverse and vertically structured, but their spatio-temporal dynamics along the water column as well as physico-chemical parameters and interactions with other bacterial species that drive the community assembly have so far not been explored in depth. Here, we present a detailed investigation of the MOB and bacterial community composition and a large set of physico-chemical parameters in a shallow, seasonally stratified, and sub-alpine lake. Four highly resolved vertical profiles were sampled in three different years and during various stages of development of the stratified water column. Non-randomly assembled MOB communities were detected in all compartments. We could identify methane and oxygen gradients and physico-chemical parameters like pH, light, available copper and iron, and total dissolved nitrogen as important drivers of the MOB community structure. In addition, MOB were well-integrated into a bacterial-environmental network. Partial redundancy analysis of the relevance network of physico-chemical variables and bacteria explained up to 84% of the MOB abundances. Spatio-temporal MOB community changes were 51% congruent with shifts in the total bacterial community and 22% of variance in MOB abundances could be explained exclusively by the bacterial community composition. Our results show that microbial interactions may play an important role in structuring the MOB community along the depth gradient of stratified lakes.

## Introduction

Atmospheric concentrations of the potent greenhouse gas methane have steadily increased since the pre-industrial era from 722 to 1874 ppb.^1^ Freshwater lakes are significant natural methane sources, responsible for about 70% of the freshwater methane emissions (DelSontro et al., 2018; Sanches et al., 2019). Methane is primarily generated in their sediments by methanogenesis, but to a smaller extent, methane production can also occur in the oxic epilimnion of lakes *via* different suggested pathways (Grossart et al., 2011; Bogard et al., 2014; Tang et al., 2016; Bižić-Ionescu et al., 2018; Günthel et al., 2019). The magnitude of the methane fluxes to the atmosphere is under strong control of anaerobic and aerobic methane oxidation (Chistoserdova, 2011; Nordi et al., 2013; Graf et al., 2018; Martinez-Cruz et al., 2018). Aerobic methane-oxidizing bacteria (MOB) oxidize up to 90% of methane within the water column of freshwater systems (Bastviken et al., 2003, 2008). They mainly belong to *Gammaproteobacteria* and *Alphaproteobacteria* and are often referred to as type I and type II MOB, respectively (Hanson and Hanson, 1996; Chistoserdova and Lidstrom, 2013; Knief, 2015).

Freshwater lakes represent complex spatially and temporally structured environments that determine the composition of MOB assemblages and their activity in numerous ways. Temperate lakes usually develop a thermal stratification during summer months, under which anoxia can develop in the bottom waters allowing large amounts of methane to accumulate (Schubert et al., 2012). During this phase, the highest MOB activity can be detected at the bottom part of the oxycline, where methane and oxygen counter gradients meet (Bastviken et al., 2002; Sundh et al., 2005; Borrel et al., 2011). Such methane oxidation zones migrate within the water column as stratification progresses (Carini et al., 2005). Competitiveness under specific oxygen, methane, copper and iron concentrations can be a factor leading to MOB niche differentiation (Knief, 2015; Chidambarampadmavathy et al., 2017; Guggenheim et al., 2019; Mayr et al., 2019; Reis et al., 2020). This is thought to be partially coupled to the composition and property of the expressed methane monooxygenase (MMO), which initiates the methane oxidation process and exists in two main forms (Sirajuddin and Rosenzweig, 2015). The particulate MMO (pMMO) is a copper-dependent enzyme with high methane affinity but slower turnover rate (Lee et al., 2006; Ross et al., 2019). It appears to be almost ubiquitous among MOB (Knief, 2015). The soluble MMO (sMMO) uses iron in its active center and is only found in some MOB (Merkx et al., 2001; Tinberg and Lippard, 2011). In addition to the mentioned parameters, other physico-chemical variables can shape the MOB abundance, their community structure and ecosystem function. For instance, low water temperature and nitrogen-rich conditions favor the growth of type I over type II MOB (Tsutsumi et al., 2011; He et al., 2012a; Siljanen et al., 2012). Furthermore, lanthanides have been shown to be essential in the methane metabolism by MOB (Pol et al., 2014; Picone and Op den Camp, 2019). The so-called “lanthanide switch” induces the upregulation of the more efficient methanol dehydrogenase (MDH), which oxidizes methanol produced by MMO and may have a significant effect on MOB activity and their community composition (Vu et al., 2016; Yu and Chistoserdova, 2017; Chistoserdova, 2019). Methane oxidation by MOB under anoxic conditions has recently been shown to be important in stratified lakes (Deutzmann et al., 2014; Oswald et al., 2016a,b; Graf et al., 2018; van Grinsven et al., 2020). The relative importance of anaerobic to aerobic methane oxidation rates can differ due to given physico-chemical environmental conditions, such as the availabilities of nitrate, nitrite, sulfate, manganese, or iron within different depth zones (Oswald et al., 2016b; Roland et al., 2018; van Grinsven et al., 2020).

Apart from physico-chemical parameters, co-occurring organisms can also affect the MOB community composition directly or indirectly (Stein, 2020). Heterotrophic richness has been shown to enhance methane oxidation activity by MOB (Ho et al., 2014), as accompanying organisms can either remove inhibiting substances (e.g., methanol) or provide stimulating factors (e.g., essential nutrients such as cobalamin; Stock et al., 2013; Iguchi et al., 2015; Veraart et al., 2018; Singh et al., 2019). On the other hand, MOB also select for certain heterotrophs by providing organic metabolites (e.g., acetate) or by removing toxic compounds (e.g., formaldehyde; Morris et al., 2013; van der Ha et al., 2013; Oshkin et al., 2015; Gilman et al., 2017; Xing et al., 2018). Indeed, MOB play an integral role in transferring methane-derived carbon and other metabolites to the microbial pool and higher trophic levels of the food web (Jones and Grey, 2011; Sanseverino et al., 2012; Agasild et al., 2014). There is thus considerable evidence that ecological interactions can be important drivers in shaping the MOB community composition, but this hypothesis has so far not really been studied in the context of environmental data.

In this study, we investigated the combined effects of microbial communities and the physico-chemical environment on MOB community assemblies in order to improve our understanding of MOB-based ecosystem function (i.e., methane removal) under varying conditions. As the short literature review above has demonstrated, the potential drivers of MOB diversity and abundance are highly complex. Yet, few studies have so far been conducted that tried to systematically identify important drivers among the large set of potential factors. Over several years, we assembled a unique dataset of vertical profiles of the total bacterial and MOB community and a large set of physico-chemical variables in a eutrophic, seasonally stratified, and sub-alpine lake (Rotsee). Statistical analysis was used to link spatio-temporal fluctuations of physico-chemical variables and bacterial community composition to MOB abundance data with the goal of identifying key drivers of the MOB community structure. In particular, we aimed to determine the extent to which bacterial interactions vs. physico-chemical drivers determine the MOB community structure.

## Materials and Methods

### Site Description, *in-situ* Profiling, Sample Collection, and Analysis

Rotsee is a small (0.5 km^2^), eutrophic lake located in central Switzerland with a maximum depth of 16 m. Its wind-shielded location allows stable stratification from spring until mid to late autumn with an oxycline usually developed between 6 and 9 m depth. During stratification methane accumulates in the anoxic water column and reaches concentrations up to 1 mM (Schubert et al., 2010; Oswald et al., 2015). Sample collection was conducted close to the deepest point of the lake during 3 consecutive years at the beginning of stratification (June 2013), during peak stratification (August 2013), and shortly before the lake overturns (September 2014 and September 2015). Detailed methods of physico-chemical profiling, sampling, and analysis are reported in the Supplementary Material. Our physicochemical dataset includes the following parameters: conductivity (Cond), turbidity (Turb), depth (pressure), temperature (T), pH, photosynthetically active radiation (PAR, herein equated as light), concentrations of oxygen (O_2_), chlorophyll a (Chl-a), total sulphide (S_Tot_ = H_2_S, HS^−^, S^2−^), dissolved organic carbon (DOC), total dissolved nitrogen (TDN), dissolved inorganic carbon (DIC), nitrite (NO_2_), nitrate (NO_3_), ammonium (NH_4_), sulphate (SO_4_), phosphate (PO_4_), dissolved (M_Diss_) and total (M_Tot_) metal concentrations [copper (Cu), iron (Fe), manganese (Mn), zinc (Zn), chromium (Cr)], methane (CH_4_) as well as ^13^C/^12^C isotopic ratio of CH_4_ (δ^13^C-CH_4_). Particulate metal (M_Part_) concentrations were obtained by subtracting dissolved metal from total metal concentrations. Bioavailable metal fractions (M_DGT_) were measured *via* the Diffusive Gradients in Thin film (DGT) technique (Davison, 2016). Detailed information on the retrieval of the DGT data can be found in (Guggenheim et al., 2019).

### DNA Sampling and Extraction, *pmoA* qPCR, Library Preparation, and Sequencing

Bacterial DNA was obtained by filtration of water samples and extraction from 0.2 μm polycarbonate filters using the PowerWater® DNA Isolation Kit (MoBio Laboratories, Carlsbad, CA, USA). Further details on DNA sampling and processing are reported in the Supplementary Material. The 16S rRNA and *pmoA* genes served as marker genes for the total bacterial community and MOB detection, respectively (Dumont, 2014). Quantitative polymerase chain reaction (qPCR) on the *pmoA* gene was conducted using the primer pair A189f and mb661r (5′-GGNGACTGGGACTTCTGG-3′, 5′-CCGGMGCAACGTCYTTACC-3′, Eurofins Genomics, Ebersberg, Germany), which covers most alphaproteobacterial and gammaproteobacterial MOB (Costello and Lidstrom, 1999). Although this primer set is specific for *pmoA*, it disfavors alphaproteobacterial MOB (Bourne et al., 2001). Verrucomicrobial *pmoA* sequences and sequences of NC10 phylum were not covered by the applied primer pair. *pmoA* amplicon libraries were prepared using the above-mentioned primers with Illumina Nextera overhang sequences at the 5′-end (Microsynth AG, Balgach, Switzerland). Amplicons were purified using AMPure XP beads (BeckmanCoulter Inc., Fullerton, CA, USA). Indexing and sequencing (MiSeq platform, Illumina Inc., San Diego, CA, USA) were conducted by the Genomics Facility Basel (Basel, Switzerland). Library preparation and Illumina sequencing of 16S rRNA genes were performed by Microsynth AG (Balgach, Switzerland). 16S rRNA genes were amplified using the primers S-D-Bact-0341-b-S-17 (5′-CCTACGGGNGGCWGCAG-3′, 341F) and S-D-Bact-0785-a-A-21 (5′-GACTACHVGGGTATCTAATCC-3′, 805R; Herlemann et al., 2011). Anaerobic methanotrophic archaea (ANME) or related archaea were excluded from the analysis as previous research showed they are of minor importance in the Rotsee water column (Oswald et al., 2015).

### Sequence Processing, Phylogenetic Analysis, and Data Deposition

16S rRNA gene and *pmoA* sequencing data were analyzed by the Genomic Diversity Centre (GDC, Zurich, Switzerland). Raw data were quality controlled using FastQC (v0.11.4) and MultiQC (v0.7). 16S rRNA gene low quality ends of reads were trimmed with PRINSEQ-lite (v0.20.4) and merged using usearch (v8.1.1812_i86linux64). *pmoA* reads were trimmed and merged using usearch (v9.2.64_i86linux 64) and FLASH (v1.2.11), respectively. Merged reads were primer-site trimmed by cutadapt v1.5 and v1.12, respectively. PRINSEQ-lite (v0.20.4) was used to filter and size-select the amplicons. Sequences were clustered into operational taxonomic units (OTUs) at a 97% similarity cut-off level using the uparse (16S rRNA gene) and an 86% similarity cut-off level using the unoise (*pmoA*) workflow with usearch (Wen et al., 2016). Taxonomic assignment of the representative sequences was set using the UTAX classifier together with the GreenGene database (May 2013) for 16S rRNA gene, and the SINTAX classifier with the *pmoA* database from (Wen et al., 2016). All sequence data have been deposited at the ENA database under the accession numbers PRJEB28460 (16S rRNA gene) and PRJEB28505 (*pmoA*).

### Depth Zones and Statistical Analysis

**Figure 1:**
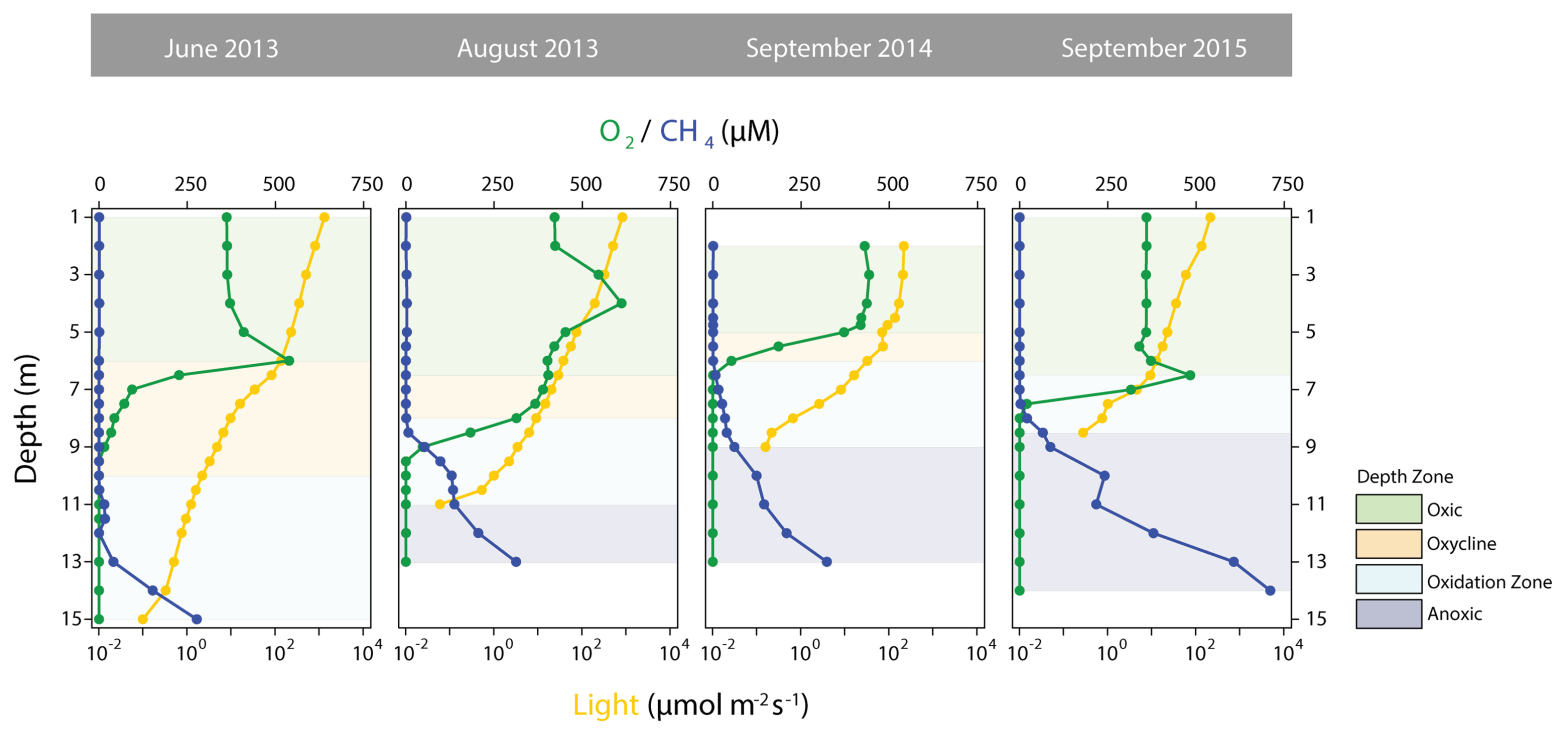
Depth profiles of selected physico-chemical parameters distinguishing the depth zones in Rotsee. Oxygen (O_2_) concentrations are shown in green, methane (CH_4_) concentrations in blue and photosynthetically active radiation (light, logarithmic scale) is depicted in dark yellow. Green shaded areas denote the oxic zones, yellow the oxyclines, blue the oxidation zones, and dark blue the anoxic zones.

As a basis for ecological interpretation and statistical analysis, we divided the water column into four zones with contrasting environmental conditions (Figure 1). The “oxic” zone ranged from the surface to depths where O_2_ started to decrease and where CH_4_ remained stable at low concentrations, but epilimnetic CH_4_ sources might be present (Hofmann et al., 2010; Donis et al., 2017). This zone was followed by the “oxycline” zone, where O_2_ was present but declined with depth, while CH_4_ remained low. The lower boundary of the oxycline zone ended where CH_4_ accumulation began. Below the oxycline, O_2_ was below detection, but in Rotsee *in-situ* photosynthetic O_2_ production still provides oxygen (Oswald et al., 2015). The “oxidation zone,” in which CH_4_ was mainly consumed, was thus the zone from where CH_4_ began to increase and either O_2_ was present at measurable concentrations or light levels (PAR) allowed for photosynthesis. Therefore, this zone ended where PAR fell below detection limit. The dark bottom water was defined as the “anoxic” zone, where O_2_ for aerobic CH_4_ oxidation was lacking.

Statistical analysis was performed within the R statistical software environment (version 3.4.3; R Core Team, 2020) using the vegan, phyloseq, mixOmics, EcoSimR, igraph, and picante packages (Csárdi and Nepusz, 2006; Kembel et al., 2010; McMurdie and Holmes, 2013; Gotelli et al., 2015; Rohart et al., 2017; Oksanen et al., 2018). 16S rRNA gene (bacterial community) and *pmoA* (MOB) reads occurring less than 3 or 50 times in at least three samples, respectively, were removed from the OTU tables. To compare relative OTU abundances, reads were standardized to the mean sequencing depth. 16S rRNA gene data assigned to MOB species was excluded in statistical models incorporating the *pmoA* data set to avoid redundant correlations. Alpha diversity measures were calculated using default vegan functions (richness, Shannon). To obtain profiles for comparison and for co-linearity assessment, a number of missing values were imputed using the missMDA package [10 complete profiles for NO_2_, NO_3_, NH_4_, SO_4_, PO_4_, DIC (all June 2013), Chl-a (September 2014 and 2015), δ^13^C-CH_4_ (June 2013, September 2015), and eight missing values for pH in September 2014; star labeled plots in Supplementary Figure S1; Josse and Husson, 2016]. Except for pH, the imputed profiles were not incorporated in the statistical models but are discussed in the context of their co-linearity with non-imputed variables. Predicted values were tested by multiple‐ and over-imputation (Supplementary Figure S2).

A principal component analysis (PCA) with the environmental variables was performed to summarize the changes in physico-chemical variables along the depth gradients during temporal succession. Pearson correlations with Holmes-corrected *p*-values of physico-chemical parameters were calculated to assess the influence of single physico-chemical variables on specific MOB species. The mvabund package was used to test environmental, total bacterial and MOB community structure differences between different depth zones (Wang et al., 2012). To assess the environmental influence on the MOB community structuring, we applied canonical correlation analysis (CCA) based on selected environmental parameters (forward, backward, and both, incorporating variables that were chosen at least in two selection strategies). Variables with variance inflation factors (VIFs) > 10 were removed prior to model selection and significance was assessed by permutation tests (9,999 permutations).

Randomness in co-occurrence of MOB and total bacterial community was tested with C-score metric and quasiswap algorithm in a null model using the EcoSimR package (Gotelli and Ulrich, 2012; Gotelli et al., 2015). To determine if phylogenetically related species cluster within specific sites in different depth zones, we assessed the standardized (z-score) effect size (SES) of mean pairwise distances (MPD) and the mean nearest taxon distances (MNTD; Webb et al., 2002). SES were compared to a null model (“richness” null model, 999 randomizations of the phylogenetic tree) and differences between zones SES were assessed by ANOVA after checking for variance homogeneity and normality of the data distributions, followed by Tukey’s HSD. MPD is sensitive to differences in phylogenetically more distant taxa, whereas MNTD is sensitive to differences of phylogenetically more closely related taxa (i.e., tip of a phylogenetic tree). Non-metric multidimensional scaling (NMDS) of Bray-Curtis dissimilarity matrix of the square root transformed Wisconsin standardized OTU tables (bacteria and MOB) were determined. Overall similarity of MOB and the total bacterial community was assessed by Procrustes analysis of the first two dimensions of the respective NMDS plots. Significance of the Procrustes was estimated by a correlation-like statistic based on the squared m12 algorithm (9,999 permutations; Peres-Neto and Jackson, 2001).

To evaluate the inter-correlations of the total bacterial community, MOB and environmental variables explaining the depth zonation, we used the supervised N-integration discriminant analysis with DIABLO from the mixOmics package in R (Rohart et al., 2017). This analysis extracts complementary information from several data sets measured on the same samples but across different data type platforms (i.e., *pmoA*, 16S rRNA gene, and environmental variables) and aims to understand the interplay between the different levels of data that were measured. Optimal sparsity parameters were determined by computing M-fold cross-validation scores. The relative influence of inter-correlations of the selected environmental variables and bacterial community members on MOB at an association relevance level > 0.5 was assessed by partial Bray-Curtis dissimilarity-based redundancy analysis using Hellinger standardized 16S rRNA gene and *pmoA* data and scaled and centered environmental data. Variables having a VIF > 5 (environmental data) and VIF > 3 (16S rRNA gene) were stepwise removed and selected (forward, backward, and both) prior to analysis. The results of the DIABLO approach were analyzed by building relevance networks visualized with Cytoscape 3.7.2 using the EClerize layout. Non-randomness of the networks was tested by comparing the network to 10,000 random Erdös-Réyni networks with similar numbers of edges and nodes (Ju et al., 2014; Weiss et al., 2016). The network GML files are available as Supplementary Materials. See also section Supplementary Material for additional statistical analysis and plots.

## Results

### Limnological Conditions and Zonation

**Figure 2:**
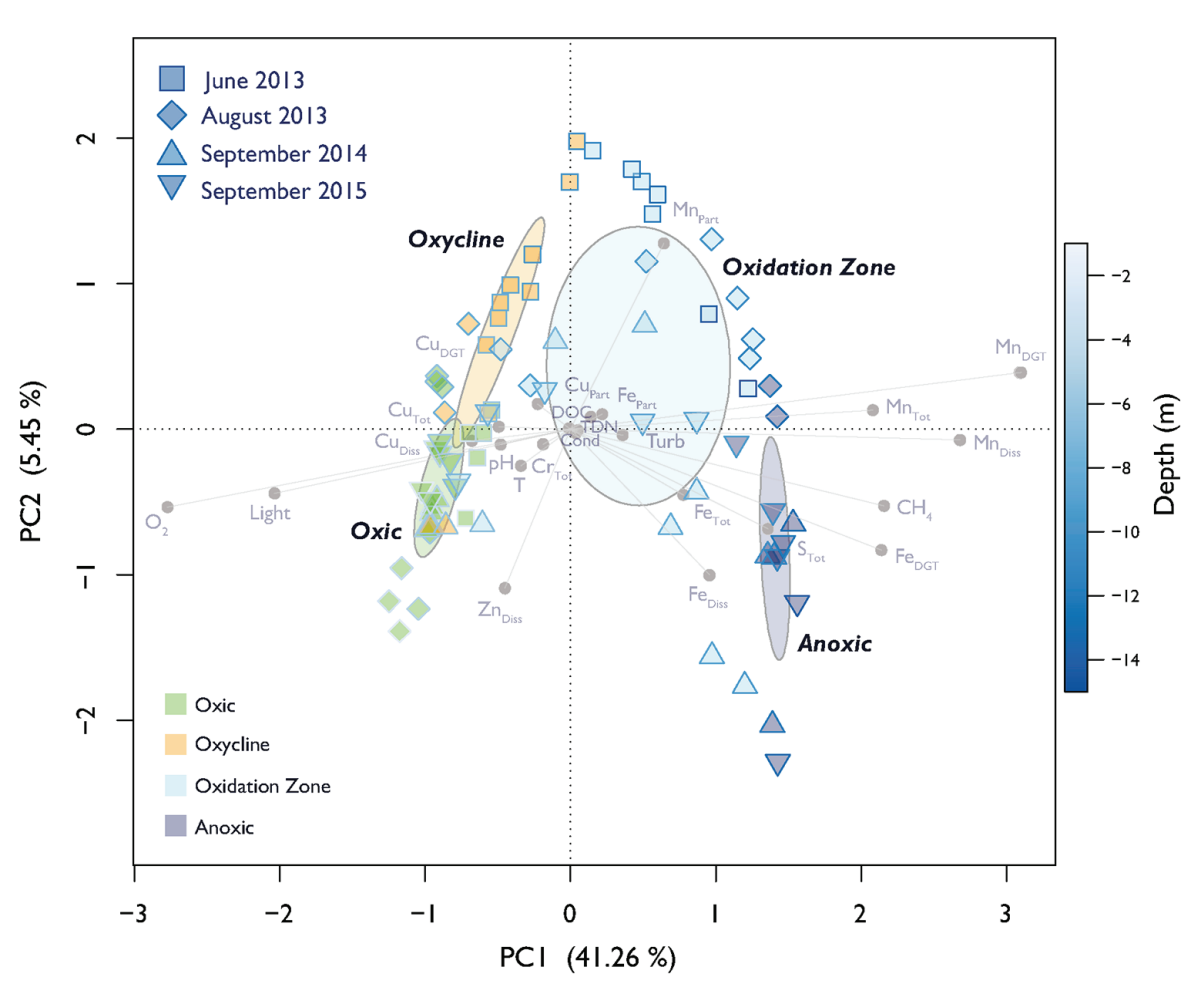
Principal component analysis (PCA) of physico-chemical variables. Dispersion ellipses depict the standard error of weighted average scores of depth zone groupings (confidence limits = 0.95). Symbols show scores of individual samples. The sampling date and assigned depth zones are colored accordingly. The color of the symbol outline illustrates the depth of the respective sample according to the depicted color gradient. The color of the symbol filling encodes the affiliation to the specific depth zone. Environmental variable loadings are depicted in light gray. Explained variance of PC1 and PC2 are given in parenthesis.

The defined depth zones over all four field campaigns were consistently different from each other in terms of their overall physico-chemical properties (mvabund: Likelihood Ratio Test (LRT) = 778.8, *p* < 0.001; Figure 1), and sampling time contributed considerably to the variability in the dataset (see ordination of samples based on their physicochemical characteristics in Figure 2). The annual T-driven stratification during summer months resulted in a narrowing of the oxycline and the oxidation zone as the season progressed (see T(°C) profiles in Supplementary Figure S1). The surface water was always well-oxygenated and O_2_ concentrations fell below detection limit usually in the upper half of the oxidation zone (Figure 1). The anoxic zone was substantially enriched with CH_4_, while only small amounts of CH_4_ (0.12–1.07 μM) were detected in the oxic zone, which, however, was still oversaturated relative to the atmosphere (Schubert et al., 2010). In September 2015, the oxycline was combined with the oxidation zone as CH_4_ profiles already slightly increased, where O_2_ decreased. In June 2013, light was detected almost to the sediment, therefore an anoxic zone was not defined. Vertical profiles of additionally measured physico-chemical parameters are summarized in Supplementary Figure S1. Turbidity (Turb) maxima were mostly congruent with Chl-a throughout the water column as profiled in June and August 2013. The anoxic zone exhibited substantial concentrations of TDN, PO_4_, DIC, and reduced substances (Fe_DGT/Diss_, Mn_DGT/Diss_, S_Tot_, and NH_4_). Cu_DGT_, Cu_Diss_, and Cu_Tot_ were found to be highest at the lake’s surface and decreased strongly in the lower oxic zone and in the oxycline, whereas Cu_Part_ concentrations usually peaked within the oxidation zone. Some variables exhibited pronounced co-linearity (see vectors in PCA Figure 2; Supplementary Figure S2).

### Microbial Community Structure (16S rRNA Gene)

**Figure 3:**
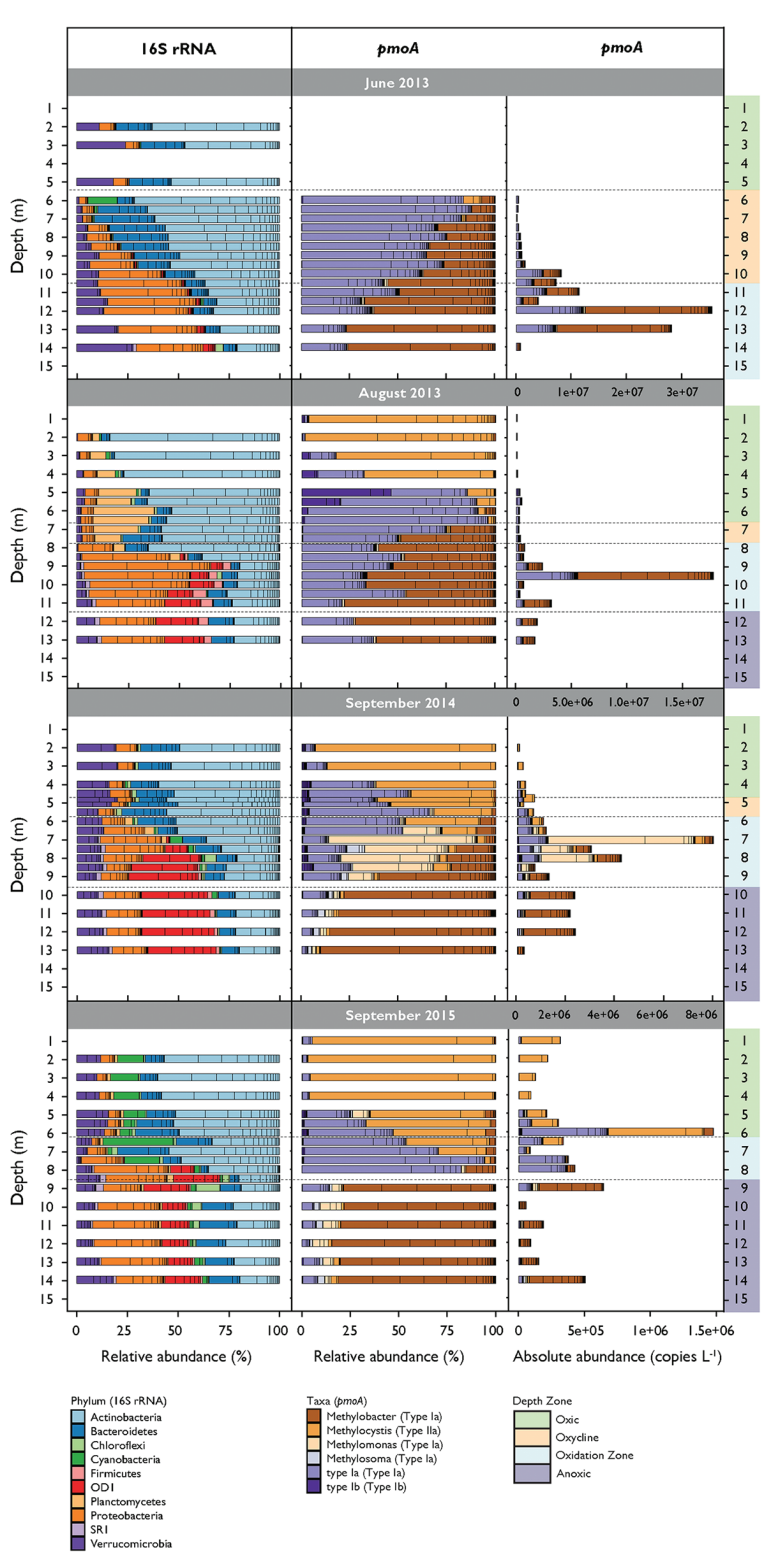
Relative abundance of most abundant bacterial phyla (16S rRNA gene) and methane-oxidizing bacteria (MOB) genera (*pmoA*) along the depth gradient during the different sampling dates. Bacteria phyla represent sequence reads that occur at least in 10% of the samples and represent at least 1% of total read counts. Bacterial and MOB relative abundances are standardized to the mean sequencing depth. MOB absolute abundances are calculated as relative abundance multiplied by *pmoA* copy numbers per liter determined by quantitative polymerase chain reaction (qPCR).

An average of 41,951 reads per sample were assigned to 1,829 unique bacterial OTUs after filtering. Alpha diversity over all campaigns increased from around 400 OTUs in the surface water to approximately 1,000 OTUs close to the sediment (see alpha diversity plots in Supplementary Figure S3). Sixteen OTUs were assigned to proteobacterial MOB and five OTUs to potential verrucomicrobial MOB. Together they represented 1.07% of all filtered reads. These OTUs were removed in analysis for co-occurrence that incorporated both 16S rRNA gene and *pmoA* data to avoid trivial correlations. Actinobacteria, Bacteroidetes, non-MOB Proteobacteria, and Verrucomicrobia dominated the communities during all campaigns (Figure 3). Actinobacteria and Proteobacteria abundance tended to decrease or increase with depth, respectively. Cyanobacteria were abundant in both the oxic zone and oxycline in September 2015 and were present in lower proportions in the other three campaigns. Firmicutes were detected in the oxidation and anoxic zone. OD1 were highly abundant in the oxidation and anoxic zone in August 2013 and in September 2014 and 2015. Planctomycetes inhabited the oxic zone and oxycline mainly in August 2013. Microbial community structures were different between the sampling dates (mvabund: LRT = 29,741, *p* < 0.001) and not randomly distributed among depths over all campaigns (C-score metric for randomness in co-occurrence = 14.16, *p* < 0.001, SES = 24.75). Bacterial communities were further structured along the depth gradient during all campaigns and were significantly different between the depth zones (mvabund: LRT = 45,650, *p* < 0.001; see NMDS ordination in Supplementary Figure S4).

### MOB Community Structure (*pmoA*)

We obtained an average of 89,816 *pmoA* reads per sample, which resulted in 121 OTUs after removing sparse OTUs (i.e., less than 50 reads in at least three samples). Alpha diversity increased with depth from 21 OTUs in the surface waters to 110 OTUs in deeper waters (Supplementary Figure S3). Sequences from type I (*Gammaproteobacteria*) and type II (*Alphaproteobacteria*) MOB were identified (Figure 3). Type I MOB were assigned to *Methylobacter*, *Methylomonas*, *Methylosoma*, and various environmental clusters (type Ia and type Ib), whereas type II MOB comprised only *Methylocystis*. MOB communities were structured along the depth gradient and differed between the depth zones and campaigns (mvabund: LRT = 3,134/2,900, *p* < 0.001; see NMDS ordination in Supplementary Figure S5). June 2013 was dominated by *Methylobacter* and type Ia (herein, we refer to type Ia excluding *Methylobacter*, *Methylomonas*, and *Methylosoma*) predominantly inhabiting the lower oxycline and the oxidation zone (Figure 3). *Methylobacter* was abundant in August 2013 in the oxidation and anoxic zone, whereas type Ia occurred from the oxycline on downward. In September 2014 and 2015, *Methylobacter* was also detected within anoxic waters. *Methylocystis* was found mainly in the oxic part of the lake, with the highest relative abundance in September 2015. *Methylomonas* and *Methylosoma* were most abundant in the CH_4_ oxidation zone of September 2014. Type Ib MOB were detected in low numbers in August 2013 in the lower part of the oxic zone and could also be found more dispersed within the oxic zone and the oxycline in September 2014 and 2015.

**Figure 4:**
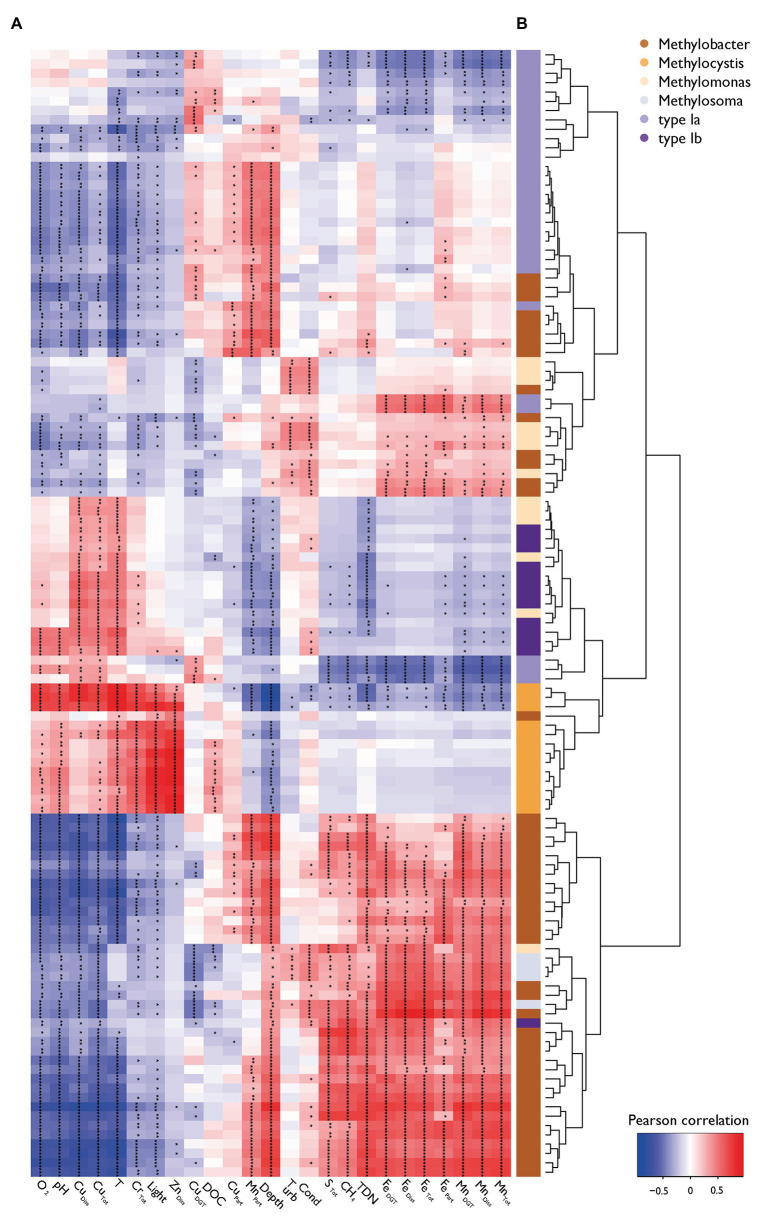
Pearson correlation heatmap of MOB (*pmoA*) and environmental variables. **(A)** The heatmap of the Pearson correlation of specific MOB OTUs abundance with physico-chemical variables is ordered by its column and row means. Asterisks indicate levels of Holmes corrected values of *p* (^*^ *p* < 0.05, ^**^ *p* < 0.01, ^***^ *p* < 0.001). **(B)** A hierarchical environmental clustering dendrogram of MOB and their color coding according to their taxonomic affiliation are depicted on the right.

Statistical testing confirmed that MOB communities were not randomly distributed within the water column (C-score = 118.93, *p* < 0.001, SES = 98.4) and showed phylogenetic relatedness higher than expected by chance in each sample within the different zones (SES of MPD < 0, SES of MNTD < 0, SES are shown in Supplementary Figure S6). Phylogenetic relatedness at higher node levels changed between specific samples in the oxycline compared to the anoxic zone [ANOVA *F*(3,66) = 3.70, *p* < 0.05, Tukey’s HSD < 0.01], whereas phylogenetic relatedness at the tree-tip level (i.e., lower node levels) was highest within the oxidation and anoxic zone [ANOVA *F*(3,66) = 7.17, *p* < 0.001, Tukey’s HSD < 0.01]. These statistics indicate that MOB clades that are phylogenetically highly similar coexist within specific sites in the oxycline, whereas phylogenetically less similar MOB tend to be mutually exclusive. Inversely, sites within the anoxic zone have a narrower phylogenetic structuring but highly similar MOB tend to be mutually exclusive. Broad phylogenetic correlation with environmental structuring is evident, but some MOB show environmental preferences that are distinct from their close phylogenetic relatives (see color coded bar for genus in Figure 4).

### Identifying Environmental and Microbial Drivers of MOB Communities

**Figure 5:**
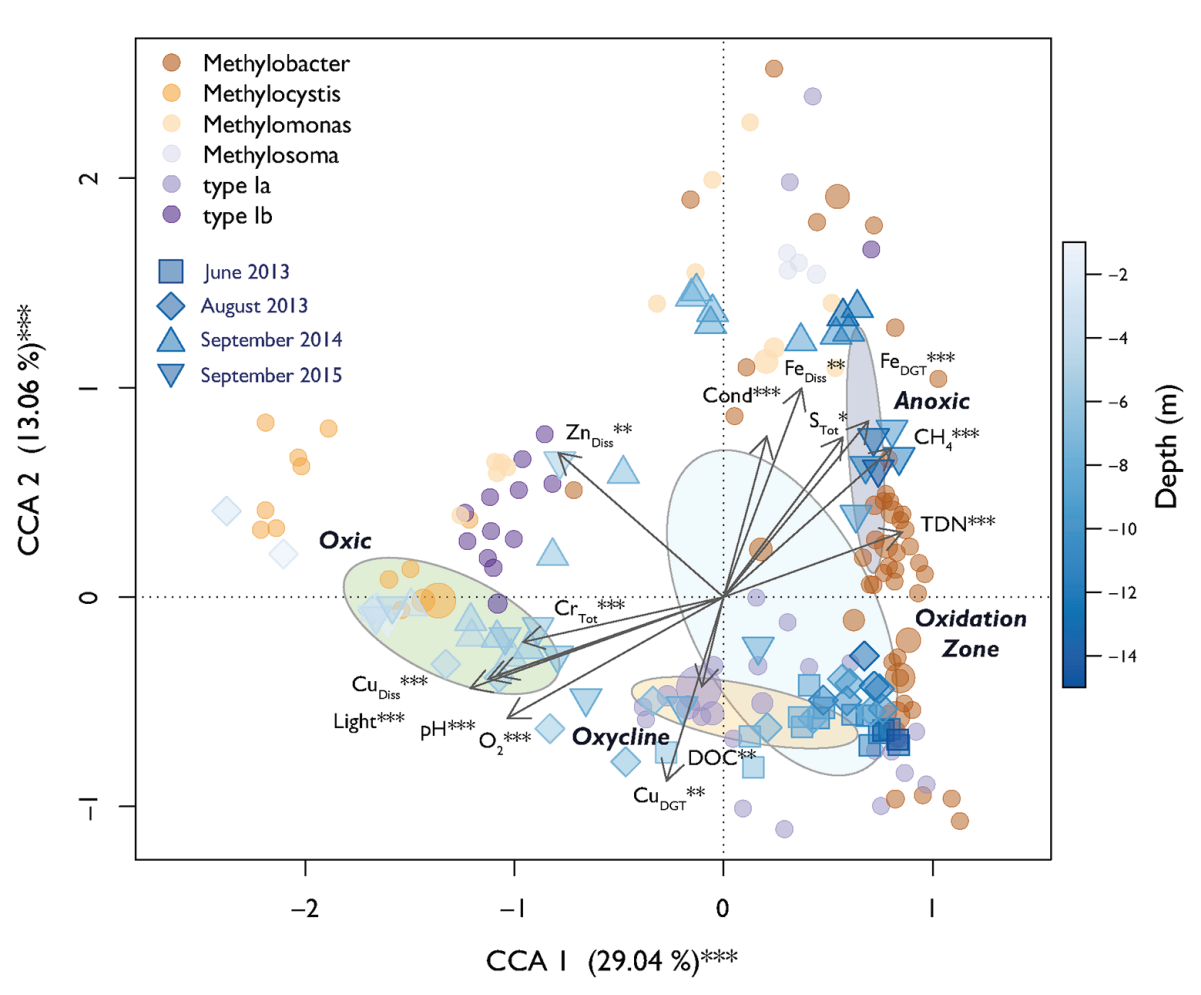
Canonical correspondence analysis (CCA) biplot of relative MOB (*pmoA*) abundance. Dots indicate specific MOB OTUs and are colored according to their taxonomic affiliation. The diameter of the dots is relative to the square root of the sums of the read counts standardized to the mean sequencing depth. Squares and triangles depict scores of specific sampling depths during different sampling dates. The fill color declares the depth of the respective sample according to the depicted color gradient. Dispersion ellipses show standard errors of weighted average scores of depth zones (confidence limits = 0.95). Environmental variables are fitted as arrows and the explained variance for canonical correlation analysis (CCA) axes 1 and 2 are given. Asterisks represent significance of permutational ANOVAS of the single variables and axes (^**^ *p* < 0.01, ^***^ *p* < 0.001).

A parsimonious CCA based on selected environmental variables explained a total of 69% of the MOB community structure over all campaigns with 42% of variation being explained on the first two axes (Figure 5). CH_4_ and O_2_ were the expected strong antipodal drivers. Light, Cu_Diss_, pH, Fe_DGT_, and TDN also contributed to the first canonical axis. Procrustes analysis showed statistically significant similarity between structuring of MOB and the rest of the bacterial community along the depth gradients over all sampling dates (Procrustes correlation = 0.51, *p* < 0.001). This could indicate either biological interactions between MOB and other microbes or similar niche preferences.

**Figure 6:**
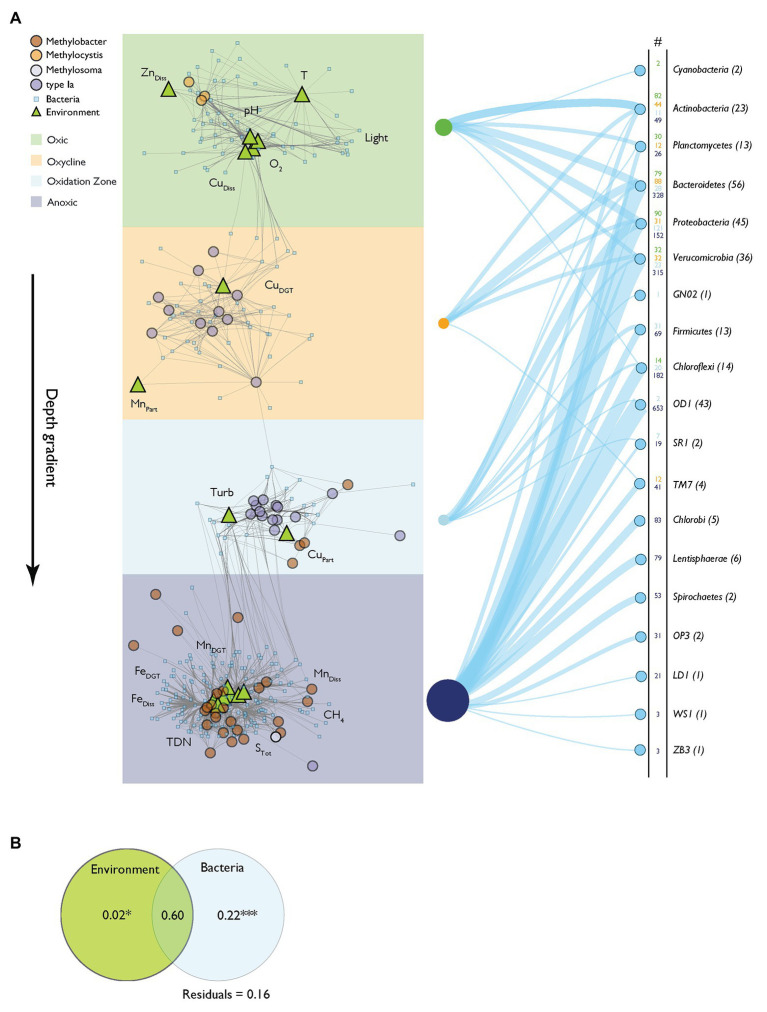
Relevance network based on N-integration discriminant analysis. **(A)** Relevance network of positive correlations between MOB, bacteria and physico-chemical variables. The network architecture reflects the assigned depth zones depicted as colored backgrounds. Gray network edges represent association relevance > 0.5. Nodes use symbols and color according to their source group: MOB (*pmoA* OTUs) are depicted as circles colored according to their phylogenetic group. Environmental variables are annotated as light green triangles. Bacterial 16S rRNA gene operational taxonomic units (OTUs) are shown as small, light blue squares. The right side of the panel shows the connectivity of distinct bacterial phyla to MOB within the different depth zones of the network. The numbers are colored according to the depth zones and connections are depicted as blue lines having relative widths according to the numbers of connections. Numbers in parentheses show the total numbers of specific bacterial phyla found in the network. **(B)** Partial redundancy analysis of model selected associated network variables (bacteria and physico-chemical variables) assessing influence on MOB occurrence. Significance levels of testable fractions are annotated (^***^ *p* < 0.001).

Network analysis provides a framework to study associations between several classes of variables (Barberán et al., 2012; Comte et al., 2016). We constructed a relevance network to analyze the connection of MOB and the bacterial community composition (*pmoA* data and 16S rRNA gene data excluding 16S rRNA gene MOB OTUs from the latter) and physico-chemical variables (Figure 6A, for network characteristics see Supplementary Table S1). Fifty-nine MOB, 271 bacterial OTUs, and 17 physico-chemical variables formed distinct networks, with sub-networks that conformed to the depth zonation of Rotsee. This confirms that the originally hypothesized zonation can be broadly reconstructed from the dataset. The network architecture showed positive inter-correlations of *Methylocystis* with O_2_, Cu_Diss_, light, pH, T, and Zn_Diss_ in the surface waters (oxic zone). Type Ia MOB in the oxycline were connected to Mn_Part_ and Cu_DGT_, and with Cu_Part_ and Turb in the oxidation zone. Within greater depths (anoxic zone), *Methylobacter* was linked to a larger set of bacteria and physico-chemical variables (Mn_Diss_, CH_4_, Fe_Diss_, TDN, S_Tot_, Fe_DGT_, and Mn_DGT_). Connections of MOB with bacterial OTU were common in all depth zone subclusters and are summarized in Figure 6A (i.e., number of connections of specific phylum within the network).

We used partial redundancy analysis to distinguish the relative contributions of physico-chemical variables and bacterial interactions on the variance of MOB abundance. According to this analysis, out of a total of 84% of explained variance in MOB occurrence, 22% can be explained exclusively by bacterial interactions, but only 2% exclusively by environmental drivers (Figure 6B). Interestingly, most of the explained variance is shared by physico-chemical variables and the bacterial community (60%). Specific bacterial OTUs affiliated with MOB in the network analysis are illustrated in the Cytoscape file (Supplementary File “Relevance network.cys”).

## Discussion

MOB throughout the water column of Rotsee play a prominent role in mitigating CH_4_ emissions to the atmosphere (Schubert et al., 2010; Oswald et al., 2015). The dominance of type I MOB (*Methylobacter*, *Methylomonas*, *Methylosoma*, type Ia, and type Ib) over type II MOB (*Methylocystis*) is consistent with previously reported patterns in Rotsee and other freshwater lakes (Borrel et al., 2011; Bornemann et al., 2016; Oswald et al., 2016b; Rissanen et al., 2018; Mayr et al., 2019; Cabrol et al., 2020; Reis et al., 2020). Evidence for the presence of anaerobic MOB in Rotsee, such as NC10 (*Methylomirabilis* sp.), was not found (Mayr et al., 2019). Anyway, we identified five Methylacidiphilales in the 16S rRNA gene data set belonging to *Methylacidimicrobium cyclopophantes*, *Methylacidimicrobium tartarophylas*, and *Methylacidimicrobium fagopyrum*.

Our depth zones, defined based on coarse physico-chemical characteristics (CH_4_, O_2_, and light availability), broadly classify MOB habitat preferences during the sampling campaigns (Figures 1, 3), which confirms the broad ecological niches for MOB in stratified lakes proposed by Mayr et al. (2019). We found Rotsee to have a highly structured MOB distribution with groups of phylogenetically related MOB being present within the relatively small spatial scales of our defined depth zones. Mayr et al. previously observed MOB sharing similar depth distribution patterns in the same lake and it remained an open question how this diversity is maintained against competitive exclusion among organisms with a comparatively simple energy metabolism.

In this work, we focused on analyzing a large dataset of environmental parameters and bacterial (OTU) abundance to identify additional drivers of the MOB community structure throughout the whole stratified water column of Rotsee. This analysis suggested that in addition to CH_4_ and O_2_, also light, metal-species (Cu and Fe), pH, TDN, and S_Tot_ played a significant role in further structuring MOB communities, explaining up to 69% of community variability according to the CCA (Figure 5). These parameters additionally showed high importance in the relevance network of MOB, other bacteria and the environment (Figure 6A).

### Metals Are Important Drivers of MOB Community Composition

The availability of Cu as a co-factor of pMMO’s active site can restrict enzymatic activity in MOB communities and thus limit their growth (Semrau et al., 2010, 2018). Previous work on the role of Cu for MOB in Rotsee has already established a likely role of Cu scarcity and competition for Cu in the lake (Guggenheim et al., 2019). The present work provides additional evidence for Cu as an important factor controlling MOB community assembly: we observed positive correlations of MOB with changing importance of Cu-species along the depth gradient of Rotsee. Cu_Diss_ correlated with *Methylomonas* and type Ib OTUs, but most strongly with *Methylocystis* in the surface water (Figures 4, 5). These genera are able to use Cu acquisition mechanisms based on complexing agents to deal with low bioavailable Cu supply conditions (Ul-Haque et al., 2015). Such auxiliary peptides could mobilize Cu from the non-bioavailable Cu_Diss_ fraction (Dassama et al., 2017; Kenney and Rosenzweig, 2018). Additionally, most members of this taxon possess a high CH_4_ affinity copper-dependent pMMO isozyme, which would support oxidizing CH_4_ at the sub-micromolar CH_4_ concentrations prevalent in the surface water (Baani and Liesack, 2008; Reis et al., 2020). *Methylocystis* is often found in warmer waters (Borrel et al., 2011; Tsutsumi et al., 2011) and it is suggested that high T selects for type II over type I MOB (Sundh et al., 2005). Indeed, the warmer surface water of Rotsee seems to favor the presence of *Methylocystis*. It is thus likely that *Methylocystis* contribute to CH_4_ consumption in the oxic zone.

During late stratified periods, when CH_4_ has accumulated in the hypolimnion of Rotsee, the highest MOB abundance was found at the lower end of the oxycline and in the oxidation zone (Figure 3). Under these conditions Cu_DGT_ and Cu_Part_ correlated mostly with type Ia and *Methylobacter* OTUs (Figures 4, 5). It is thought that Cu_DGT_ is the bioavailable Cu fraction, whereas Cu_Part_ mainly represents Cu incorporated into biomass (Guggenheim et al., 2019). Cu_DGT_ concentrations decrease substantially from the oxycline toward the oxidation zone (Supplementary Figure S1). *In situ* studies focusing on the influence of Cu on MOB community structures are rare, but it has been elucidated that MOB able to express pMMO thrive even under very low levels of bioavailable Cu (< 50 nM; Cantera et al., 2016). In addition, as mentioned before, certain MOB species possess special Cu uptake mechanisms to increase the bioavailable Cu fraction.

Previous studies also reported the occurrence of *Methylobacter* species in the anoxic zones of stratified lakes (Biderre-Petit et al., 2011; Blees et al., 2014; Milucka et al., 2015; van Grinsven et al., 2020). Bioavailable and dissolved Fe (Fe_DGT_ and Fe_Diss_) correlated with most *Methylobacter* species (Figures 4, 5), although cultivated representatives are not able to express the iron-dependent sMMO enzyme (Knief, 2015) and sMMO appears to be rare in Rotsee (Guggenheim et al., 2019). However, it is possible that these MOB rely on Fe for other enzymatic pathways (e.g., formate dehydrogenase; Glass and Orphan, 2012). Furthermore, it has been suggested that the mechanism of Fe-coupled anaerobic CH_4_ oxidation is accomplished by a complex microbe-mineral reaction network in which both, MOB and iron-reducing organisms (bacteria and archaea) are directly and indirectly involved (Bar-Or et al., 2017; Cabrol et al., 2020). For example, besides methanogens being able to produce CH_4_, they are additionally involved in reducing Fe-oxides at high CH_4_ concentrations leading to intermediates, which are required by MOB for CH_4_ oxidation (Bar-Or et al., 2017). Mn_DGT_ and Mn_Diss_ also correlated with *Methylobacter* OTUs within the anoxic zone (Figures 4, 5). It has been proposed that an alternative anaerobic CH_4_ oxidation lifestyle proceeding *via* Fe(III) or Mn(IV) reduction could be relevant in CH_4_-rich anoxic zones of lakes, which could explain the increase in Fe_Diss_ and Mn_Diss_ in the deeper waters of Rotsee and its correlation with *Methylobacter* (Crowe et al., 2008; Oswald et al., 2016a; Supplementary Figure S1).

Interestingly, Zn_Diss_ was also suggested as a significant driver by our analysis and was not co-linear with other environmental variables (Figures 4, 5; Supplementary Figure S2). There was a positive correlation of *Methylocystis* with Zn_Diss_ although Zn potentially inhibits pMMO activity (Sirajuddin et al., 2014). However, the highest measured Zn_Diss_ concentrations in Rotsee were ~100 times lower than those tested experimentally by Sirajuddin et al. The close network connectivity within the oxic zone between *Methylocystis* and other bacteria (i.e., linkage to Actinobacteria, Bacteria, Proteobacteria, and Bacteroidetes; Figure 6B) might indicate indirect effects mediated by Zn. For example, the bacterial OTU1 could be assigned to the order *Candidatus* Nanopelagicus within Actinobacteria isolated from Lake Zurich (Neuenschwander et al., 2018; see also the cytoscape file). The isolate showed a reduced genome and thus might have strong metabolic dependencies on co-occurring bacteria (i.e., MOB) for lost metabolic functions that have to be provided by functional leakage (Morris et al., 2012). As Actinobacteria exhibit Zn concentration linked gene regulation mechanisms, this might mirrors a reverse effect of *Methylocystis* on this *Candidatus* Nanopelagicus (Choi et al., 2017), mediated by, i.e., the release of riboflavin, nicotinamide, and thiamine of the *Candidatus* Nanopelagicus. Anyway, further work will be necessary to confirm the role of Zn and to investigate potential mechanisms linking its availability to MOB dynamics.

### Other Environmental Controls

The highly significant contribution of total dissolved nitrogen (TDN) as an explanatory variable for MOB abundance indicates links between MOB and nitrogen availability (Figures 4, 5). In particular, TDN contributed to the position of *Methylobacter* in the relevance network (Figure 6A). The observed gradient of TDN strongly correlated with NH_4_ and exhibited similar concentration ranges suggesting that NH_4_ contributes the largest part of TDN (Supplementary Figures S1, S2). NH_4_ is a central nutrient in aquatic systems, hence a positive correlation between NH_4_ and MOB could be due to the fact that MOB assimilate NH_4_ for growth. Previously, a laboratory study with littoral wetland samples from a boreal lake has demonstrated that nitrogen load in form of NH_4_NO_3_ changed the MOB community structure and favored activity of type I MOB, particularly *Methylobacter* cells (Siljanen et al., 2012). The evolutionary linkage of the genetic sequence of *pmoA* and *amoA*, which encodes for the ammonia monooxygenase (AMO), endows most MOB, especially type I MOB, with the ability to oxidize NH_4_ through the pMMO enzyme (Knief, 2015; Khadka et al., 2018), which may be another explanation for the link between MOB and NH_4_.

CH_4_ oxidation can also be coupled directly to the nitrogen cycle. Anaerobic oxidation of CH_4_ *via* NO_2_-/NO_3_-reduction (n-damo) has normally been attributed to *Methylomirabilis* species of the NC10 phylum, which according to previous data do not appear in Rotsee (Mayr et al., 2019). However, recent whole genome and environmental metagenome analysis have revealed that various assimilatory and dissimilatory nitrogen reduction genes, such as those encoding NO_2_‐ and NO_3_-reductases, are also found among gammaproteobacterial MOB, especially *Methylobacter* and *Methylomonas* species (Chistoserdova, 2015; Zhu et al., 2016). This would suggest that NO_2_ and NO_3_ can be used by MOB to oxidize CH_4_. However, NO_2_ and NO_3_ were not detected below the oxidation zone in Rotsee (Supplementary Figure S1).

### Co-Occurrence of MOB With Bacteria and Possible Ecological Interactions

Spatio-temporal shifts in the MOB community were highly congruent with the changing composition of the total bacterial community (OTU level, Procrustes 51%). The restrictive and congruent localization of MOB and bacterial OTUs throughout the stratified periods suggests that biological interactions between MOB and other bacteria need to be analyzed in more detail.

In our network analysis we focused exclusively on positive MOB co-occurrence patterns with environmental variables and the bacterial community. This means that observed associations can arise from a number of causes: species co-occurrence can be driven by direct biological interactions such as synergism (i.e., cooperation) but also by similar niche preferences (neutral effects; Faust and Raes, 2012; Ho et al., 2016). Additionally, positive feedback loops between MOB and the total bacterial community interacting with their environment may also result in co-occurrence. It is noteworthy that positive interactions with bacteria alone (excluding impact of the environment) explained a relevant part of the MOB occurrence (22%; Figure 6B). This indicates that at least some of the correlations are through actual microbial interactions and do not just reflect overlapping niche preferences. Anyway, it is possible that these interactions are mediated through an effect chain of non-measured variables, i.e., by the influence of trophic levels ultimately influencing MOB dynamics.

However, the majority of variance in MOB abundance within the network was explained jointly by physico-chemical parameters and the bacterial community (60%; Figure 6B), while according to CCA environmental parameters explained 69% of MOB abundance. While this underscores the importance of physico-chemical parameters as drivers of the bacterial and MOB community structure, positive correlations of this type could still involve biological interactions. Physico-chemical variables may, e.g., affect the bacterial community structure, which subsequently influences the MOB community by synergistic effects or vice versa. The available data do not allow us to distinguish between these possibilities. It is further possible that unmeasured environmental variables contribute to shaping of the MOB community structure. However, it is noteworthy that bacterial and physico-chemical inter-connectivities showed high complexity in the surface layer and the hypolimnion of the lake (i.e., number of connections in Figure 6A), while inter-connectivities within the oxycline and oxidation zones were less distinct.

Hereafter, we suggest some examples of possible positive bacterial interaction mechanisms taking place in the water column of Rotsee. Within the oxidation zone, part of the type Ia and *Methylobacter* communities correlated with Turbidity (Turb), a possible indicator for primary producers as Turb showed some overlapping patterns with Chl-a depth profiles (Supplementary Figure S2). MOB can form mutualistic interactions with oxygenic phototrophs in light penetrated anoxic layers enabling CH_4_ oxidation, while potentially providing carbon dioxide in return (Milucka et al., 2015). It seems likely that these MOB clusters are the main CH_4_ consumers in Rotsee as the main aerobic CH_4_ oxidation process in Rotsee during stratification happens within the oxycline and oxidation zone and might be predominantly coupled to oxygenic primary production (Oswald et al., 2015; Brand et al., 2016). There is also evidence that CH_4_ oxidation by MOB under O_2_ limitation is indirectly connected to the reduction of alternative terminal electron acceptors by other organisms in the anoxic waters of the lake. NO_2_ and NO_3_ were not measured in the anoxic waters of Rotsee, however, NO_2_ could have been produced by Fe-/Mn-dependent anaerobic NH_4_-oxidizing bacteria, and readily used by gammaproteobacterial MOB for oxidizing CH_4_ (Ferousi et al., 2017; Kuypers et al., 2018). Due to the previously mentioned homology between pMMO and AMO, MOB and nitrifying bacteria are capable of both, CH_4_ and NH_4_ oxidation. Hence, the coexistence of MOB and nitrifying bacteria under anoxic conditions could be explained on the basis of similar niche preferences (Costa et al., 2019). Co-occurrence of MOB in the same areas as non-methanotrophic methylotrophs might be beneficial for MOB. Since high methanol concentrations inhibit MOB performance, co-occurring methylotrophs, which are able to assimilate methanol, remove this compound to levels that enable MOB to thrive (Denfeld et al., 2016). A recent *in-situ* study shows that non-methanotrophic methylotrophs induce a change in the expression of MOB MDHs *via* putative secretory compounds leading to an increased loss of methanol, which is readily taken up by the methylotrophs (He et al., 2012b; Biderre-Petit et al., 2019). For example, we found highly connected methylotrophs belonging to the family Methylophilaceae and the Verrucomicrobia subdivision 6 in the oxic zone in the relevance network. In the anoxic zone, we found members of the *S-BQ2-57* soil group belonging to Verrucomicrobia that showed high connectivity within the network. Methanol also plays a significant role between the coupling of aerobic CH_4_ oxidation and denitrification by the cooperation between MOB and denitrifying bacteria. Organic metabolites (i.e., methanol, citrate, acetate, formaldehyde, and formate) released by MOB could serve as electron donors for denitrification, where methanol is thermodynamically considered as the ideal trophic link (Kalyuhznaya et al., 2009; Zhu et al., 2016).

## Conclusion

In summary, our results indicate that the MOB community assembly in Rotsee is sensitively linked to environmental conditions and the greater bacterial community. The distinct zonation of MOB throughout the water column, which is thought to be driven by CH_4_ and O_2_ counter gradients, was additionally linked to several physico-chemical variables and their interactive effect with parts of the bacterial community. Considering the three-way relation of MOB, bacteria and environment, our analysis revealed that bacteria alone could explain significantly more of the MOB structure (22%) than the isolated physico-chemical variability (2%; Figure 6B). However, the mode of action underlying the correlations could not be unambiguously unraveled. Future studies with a strong focus on microbial interdependency that incorporate deep sequencing metagenomic and transcriptomic as well as metabolic and anabolic analysis tools will help to disentangle the mode of actions of the herein presented inter-connectivity (Zheng and Chistoserdova, 2019). Understanding the mechanisms of these biotic and abiotic interactions will help to predict the responses of MOB community functioning under diverse conditions.

## Data Availability Statement

The datasets presented in this study can be found in online repositories. The names of the repository/repositories and accession number(s) can be found at: https://www.ebi.ac.uk/ena, PRJEB28460, PRJEB28505.

## Author Contributions

CG, RF, HB, and BW planned the experiments. CG, HB, KB, and MM performed laboratory and field work. CG and RF analyzed the data. CG, RF, HB, and BW wrote the manuscript with support and input of all coauthors. All authors contributed to the article and approved the submitted version.

### Conflict of Interest

The authors declare that the research was conducted in the absence of any commercial or financial relationships that could be construed as a potential conflict of interest.

## Footnotes

^1^https://www.esrl.noaa.gov/gmd/ccgg/trends_ch4/, June 2020
