# Supplementary material for: Environmental and Microbial Interactions Shape Methane-Oxidizing Bacterial Communities in a Stratified Lake
Source: Front Microbiol. 2020 Oct 15;11:579427. doi: 10.3389/fmicb.2020.579427 (PMC7593551; doi:10.3389/fmicb.2020.579427)
Supplement: Supplementary file 1 [file Data_Sheet_1.pdf]

Environmental and microbial interactions shape methane-oxidizing bacterial communities in a stratified lake

Carole Guggenheim[1,2*#], Remo Freimann[3*#], Magdalena J. Mayr[1,2], Karin Beck[2], Bernhard Wehrli[1,2], Helmut Bürgmann[2]

[1]Department of Environmental Systems Science, Institute of Biogeochemistry and Pollutant Dynamics, ETH Zurich - Swiss Federal Institute of Technology, Zurich, Switzerland

[2]Department of Surface Waters - Research and Management, Eawag - Swiss Federal Institute of Aquatic Science and Technology, Kastanienbaum, Switzerland

[3]Department of Biology, Institute of Molecular Health Sciences, ETH Zurich - Swiss Federal Institute of Technology, Zurich, Switzerland

*Corresponding authors:
carole_guggenheim@hotmail.com; remofreimann@gmail.com

[#]These authors contributed equally to this work.

Supplementary material

Physico-chemical profiling, sampling and analysis

Sample collection was conducted close to the deepest point of Rotsee (47°04.259’N, 8°18.989’E) during three consecutive years at the beginning of stratification (June 2013), during peak stratification (August 2013) and shortly before the lake overturns (September 2014, September 2015). A custom-built ‘Profiler for *In-situ* Analysis’ (PIA) was deployed for high-resolution depth profiling and deployment of different sensors and sampling devices [1]. A multi-parameter probe (XRX 620, RBR, Ottawa, ON, Canada) recorded conductivity (Cond), turbidity (Turb), depth (pressure), temperature (T) and pH. Dissolved oxygen (O_2) was measured with normal and trace micro-optodes (types PSt1 and TOS7, Presens, Regensburg, Germany; detection limits: 125 and 20 nM). A spherical quantum sensor (LI-190 SB, LI-Cor, Lincoln, NE, USA) provided data of photosynthetically active radiation (PAR) with a detection limit of 0.1 µmol m^{-2} s^{-1}. Chlorophyll a (Chl-a) was measured in June and August 2013 using an ECO-FL fluorescence probe (Wetlabs, USA) attached to PIA.

The following variables were sampled via a rosette syringe sampler (12 x 60 ml syringes). Aliquots for total sulphide (S_{Tot} = H_2S, HS^-, S^{2-}) measurements were immediately fixed with zinc acetate (~1.3% final concentration) and determined by spectrophotometric analysis following the procedure of Cline [2]. Dissolved organic carbon (DOC) and total dissolved nitrogen (TDN) samples were analysed on a total carbon analyser (TOC-$L_{CSH/CPH}$, Shimadzu, Tokyo, Japan) after filtration (< 0.22 µm, Millex-GP polyethersulfone (PES) membrane, Millipore, Zug, Switzerland) and acidification (2M HCl; final concentration: 20 mM). Dissolved inorganic carbon (DIC) samples (August 2013, September 2014 and 2015) were filtered (< 0.22 µm) on-site and measured on the same TOC-analyser. Except for June 2013, samples for nitrite (NO_2), nitrate (NO_3), ammonium (NH_4), sulphate (SO_4), and phosphate (PO_4) were immediately filtered (< 0.22 µm), and measured by ion chromatography (881 Compact IC pro, 882 Compact IC plus, 761 Compact IC, Methrom AG, Zofingen, Switzerland) and flow-injection analysis (SAN++, Skalar, Procon AG, Burgdorf, Switzerland), respectively. Dissolved (M_{Diss}, < 0.45 µm) and total (M_{Tot}) metal samples (copper (Cu), iron (Fe), manganese (Mn), zinc (Zn), chromium

(Cr)) were acidified and quantified via inductively coupled plasma mass spectrometry (Element2, Thermo-Fisher Scientific, Reinach, Switzerland). All metal sampling equipment was acid-washed and rinsed with nanopure water before use. Particulate metal concentrations (M_{Part}) were calculated by subtracting dissolved from total metal concentrations. The Diffusive Gradient in Thin film (DGT) technique was applied to receive information about the bioavailable metal fractions (M_{DGT}) [3,4]. Details of preparation, sampling and analytical measurements of the DGT samplers are given in Guggenheim et al. [5].

Samples for dissolved methane (CH_4) concentrations were either collected with a Niskin bottle or via pumping with a gas tight tubing (PVC Solaflex, Maagtechnic, Dübendorf, Switzerland) attached to PIA. 120 ml serum bottles were filled anoxically allowing water to overflow. CH_4 oxidation was stopped by adding NaOH (pH > 12) or Cu(I)Cl and bottles were crimped airtight. Headspace injection of 20 ml N_2 was used for equilibrium and CH_4 was measured on a gas chromatograph (Agilent 6890N, Agilent Technologies, Santa Clara, CA, USA) equipped with a Carboxen 1010 column (30 m x 0.53 mm, Supelco, Bellefonte, PA, USA) and a flame ionization detector (detection limit ~0.01 µM). CH_4 concentrations in the water phase were calculated using Henry's law [6]. The same headspace was used to determine the $^{13}C/^{12}C$ isotopic ratio of CH_4 ($\delta^{13}C\text{-}CH_4$) (August 2013, September 2014) using isotope ratio mass spectrometry with a trace gas instrument (T/GAS PRE CON, Micromass UK Ltd., Wilmslow, UK) coupled to a mass spectrometer (GV Instruments, Manchester, UK; Isoprime, Stockport, UK). Data were obtained from Oswald et al. [7,8]. Isotopic ratios are given in the conventional δ-notation normalized to the Vienna Pee Dee Belemnite (VPDB) reference standard.

DNA sampling and extraction, *pmoA* qPCR and limited cycle PCR, amplicon purification

Water samples were collected either with a Niskin bottle or via pumping and were prefiltered (5.0 µm polycarbonate) and subsequently run through 0.2 µm polycarbonate membrane filters. Filters were frozen in liquid N_2 and stored at -80°C until DNA was extracted from the 0.2 µm filters using the PowerWater® DNA Isolation Kit (MoBio Laboratories, Carlsbad, CA, USA) following the

manufacturer's instructions. DNA was quantified with a NanoDrop 1000 Spectrophotometer (Thermo-Fisher Scientific, Wilmington, DE, USA).

pmoA gene numbers were quantified using the primer pair A189f (Eurofins Genomics, Ebersberg, Germany; 5'-GGNGACTGGGACTTCTGG-3') and mb661r (Eurofins Genomics, Ebersberg, Germany; 5'-CCGGMGCAACGTCYTTACC-3') [9,10] according to a protocol adapted from Henneberger et al. [11]. The qPCR reaction mixture consisted of 1 × master mix (LightCycler® 480 SYBR® Green I Master), 0.2 µM of each primer, and 2 µl of template DNA (1:10). Samples were run using the following thermal program: initial denaturation (95°C, 10 min), 10 touchdown cycles (denaturing: 95°C, 10 sec; annealing: 62-53°C (-1°C per cycle), 30 sec; extension: 72°C, 30 sec), followed by 30 cycles (denaturing: 95°C, 10 sec; annealing: 52°C, 30 sec; extension: 72°C, 30 sec). Fluorescent readings were taken at 79°C for 30 sec and melting curves at 65-97°C (0.11°C/sec). Results were analysed with the Absolute Quantification/2nd Derivative Maximum method. qPCR efficiency (E) yielded in 1.868.

For *pmoA* library preparation, triplicate PCR reactions (20 µl) were set up for each sample containing: primers (0.3 µM each, A189f, mb661r), 1 × Nebnext Q5 Hot Start HiFi PCR Master Mix (New England BioLabs, Frankfurt, Germany), and undiluted DNA extracts of the respective sample (3 µl). Cycle conditions were as follows: initial denaturation (30 sec, 98°C), 27 cycles of denaturation (10 sec, 98°C), annealing (35 sec, 54°C) and elongation (35 sec, 65°C); and final extension (5 min, 65°C). Genomic DNA from an axenic culture strain (*Methylocystis rosea* received from DSMZ, Braunschweig, Germany) served as positive control. Parallel reactions were combined and purified using AMPure XP Beads (BeckmanCoulter Inc., Fullerton, CA, USA) according to the protocol.

Additional statistical analysis

The Shapiro-Wilk test was used to assess normality of physico-chemical variables, which were subsequently log transformed if test criteria were not met. Absolute MOB abundances were calculated by applying relative abundances to *pmoA* copy numbers obtained from qPCR measurements. Non-metric multidimensional scaling (NMDS) was used to visualize temporal succession dynamics in

community structure by using the Bray-Curtis dissimilarity matrix of the square root transformed Wisconsin standardized OTU table. Differences in beta diversity among depth zones were assessed by multivariate homogeneity of dispersions [12]. Thereby, Bray-Curtis distances between samples within the depth zones are reduced to principal coordinates and distances of zone members to the zones centroid are calculated and compared by type III ANOVAs followed by Tukey's HSD (honest significant difference) test. The DIABLO relevance network at an association relevance level > 0.5 included 347 nodes and 3099 edges (Table S1).

Additional results

Light radiation was attenuated with depth and detectable down to the anoxic zones. CH_4 isotopic signatures usually became heavier from the anoxic zone towards the oxycline and shifted again to lighter signatures within the oxycline and the oxic zone (measured in August 2013, September 2014). pH decreased from the oxic to the anoxic zone and Cond slightly increased. DOC, NO_2, NO_3, SO_4, Cr_{Tot}, Zn_{Diss} either decreased from the epi- to the hypolimnion or were more or less stable throughout. Fe_{Part} and Mn_{Part} concentrations were low compared to their soluble concentrations.

16S rRNA gene beta diversity over all campaigns was smallest in the anoxic zone, and highest within the oxidation zone (ANOVA $F(3,71) = 5.50$, $p < 0.01$) reflecting a distinct community turnover in the latter. Bacteria in a sample within a specific depth zone were phylogenetically more related than expected by chance (SES MPD < 0, SES MNTD < 0), which shows the presence of ecological niches within the zonation. 16S rRNA gene based SES was highest in the anoxic zone (ANOVA $F(3,71) =$ 23.27 / 30.52, $p < 0.001$, Tukey's HSD < 0.01), which indicates lower phylogenetic relatedness within a specific site within this zone compared to the other zones. *pmoA* beta diversity did not differ between the depth zones over all sampling dates ($F(3,66) = 0.07$, $p = 0.98$) indicating a relative steady community turnover throughout the specific zones.

Supplementary figures

Figure S1. Gradients of physico-chemical parameters in Rotsee.

First panel of each plot shows June 2013, second panel August 2013, third panel represents September 2014, and the fourth panel September 2015. Background colours refer to the different depth zones (green = oxic zone, yellow = oxycline, blue = oxidation zone, dark blue = anoxic zone). A star indicates complete imputed profiles, except for pH where data points from 6.5 m onwards were imputed.

Figure S2. PCA of 10000 boot imputed datasets from the physico-chemical variables and overimputation to assess the fit of the predictive distribution.

(A) Observed values are the same from one dataset to the others whereas imputed values change. The variation of the point cloud of imputed values reflects the variability with which missing values can be predicted. The PCA shows also the co-linearity (i.e. similar directionality of arrows) for the measured variables. (B) 10000 imputations for each observed value from each variable were produced and a 90% confidence interval for each observed value was constructed using the quantiles of the overimputed values. The proportion of the other values that were missing for that observation in the original data are indicated in blue and green. Purple frames indicate variables that have missing values in the original data. Black lines show the 1:1 ratio. Units of the variables can be found in Figure S1.

Figure S3. Alpha diversity measures of 16S rRNA gene based bacterial community data (A) and *pmoA* based MOB data (B).

Figure S4. Non-metric multidimensional scaling (NMDS) of the total bacterial community structure (16S rRNA) during all sampling dates.

Grey dots depict species score of 40 detected bacterial phyla. Annotated blue squares and triangles indicate scores of individual sampling depths during different sampling campaigns (e.g., A01–A21). Fill colour represents the depth of the respective sample according to the colour gradient. Dispersion ellipses depict the standard error of weighted average scores of depth zone groupings (confidence limits = 0.95). The stress of the NMDS ordination is stated.

F**igure S5. Non-metric multidimensional scaling (NMDS) of the MOB (*pmoA*) community structure during all sampling dates.**

Blue symbols indicate scores of individual samples obtained at different sampling dates. The fill colour illustrates the depth of the respective sample. MOB OTU scores are depicted as points coloured by taxonomic affiliation. The diameter of the dots is relative to the square root of the sums of the read counts standardized to the mean sequencing depth. Dispersion ellipses represent the standard error of weighted average scores of depth zone groupings (confidence limits = 0.95). The stress of the NMDS ordination is declared.

Figure S6. Boxplots of Standard Effect Size (SES) of Mean Pairwise Distance (MPD) and Mean Nearest Taxon Distance (MNTD).

Standard Effect Sizes (SES) are compared to a null model with randomized (n = 999) tree structuring. SES-MPD and SES-MNTD of bacteria (A,B) and MOB (C,D) communities within the different depth zones are depicted. Differences in SES were tested by ANOVA followed by Tukey's HSD. Negative SES values indicate phylogenetic clustering (i.e. higher degrees of phylogenetic relatedness among species in the habitat than expected by chance) whereas positive values would represent

overdispersion. MPD is more sensitive to patterns over the complete phylogenetic tree, whereas MNTD is more sensitive to clustering and evenness of tree tips.

Table S1. Properties of the co-occurrence (DIABLO) networks compared to similarity sized Erdös-Rényi random networks.

Network characteristics (cut off)	DIABLO (0.5) / Random
Nodes	347
Edges	3’099
Average degree	17.9 / 17.8
Diameter	12 / 4
Average path length	4.77 / 2.33
Modularity	0.43 / 0.17
Modularity classes	5
Density	0.05 / 0.05
Average cluster coefficient	0.29 / 0.05
Small world coefficient	2.83

References

1. Kirf MK, Dinkel C, Schubert CJ, Wehrli B. Submicromolar oxygen profiles at the oxic-anoxic boundary of temperate lakes. Aquat Geochemistry. 2014;20: 39–57. doi:10.1007/s10498-013-9206-7

2. Cline JD. Spectrophotometric determination of hydrogen sulfide in natural waters. Limnol Oceanogr. 1969;14: 454–458. Available: http://www.jstor.org/stable/10.2307/2833813

3. Davison W. Diffusive Gradients in Thin-Films for Environmental Measurements. Davison W, editor. Cambridge University Press; 2016.

4. Menegário AA, Marques Yabuki LN, Luko KS, Williams PN, Blackburn DM. Use of diffusive gradient in thin films for in situ measurements: a review on the progress in chemical fractionation, speciation and bioavailability of metals in waters. Anal Chim Acta. 2017;983: 54–66. doi:10.1016/j.aca.2017.06.041

5. Guggenheim C, Brand A, Bürgmann H, Sigg L, Wehrli B. Aerobic methane oxidation under copper scarcity in a stratified lake. Sci Rep. 2019;9. doi:10.1038/s41598-019-40642-2

6. Wiesenburg DA, Guinasso Jr. NL. Equilibrium solubilities of methane, carbon monoxide, and hydrogen in water and sea water. J Chem Eng Data. 1979;24: 356–360. doi:10.1021/je60083a006

7. Oswald K, Milucka J, Brand A, Littmann S, Wehrli B, Kuypers MMM, et al. Light-dependent aerobic methane oxidation reduces methane emissions from seasonally stratified lakes. PLoS One. 2015;10. doi:10.1371/journal.pone.0132574

8. Oswald K, Graf JS, Littmann S, Tienken D, Brand A, Wehrli B, et al. Crenothrix are major methane consumers in stratified lakes. ISME J. 2017;11: 2124–2140. doi:10.1038/ismej.2017.77

9. Holmes AJ, Costello AM, Lidstrom ME, Murrell JC. Evidence that particulate methane monooxygenase and ammonia mooxygenase may be evolutionarily related. FEMS Microbiol Lett. 1995;132: 203–208. doi:10.1111/j.1574-6968.1995.tb07834.x

10. Costello AM, Lidstrom ME. Molecular characterization of functional and phylogenetic genes from natural populations of methanotrophs in lake sediments. Appl Environ Microbiol. 1999;65: 5066–5074.

11. Henneberger R, Chiri E, Bodelier PLE, Frenzel P, Lüke C, Schroth MH. Field-scale tracking of active methane-oxidizing communities in a landfill cover soil reveals spatial and seasonal variability. Environ Microbiol. 2015;17: 1721–1737. doi:10.1111/1462-2920.12617

12. Anderson MJ, Ellingsen KE, McArdle BH. Multivariate dispersion as a measure of beta diversity. Ecol Lett. 2006;9: 683–693. doi:10.1111/j.1461-0248.2006.00926.x

Figure S1

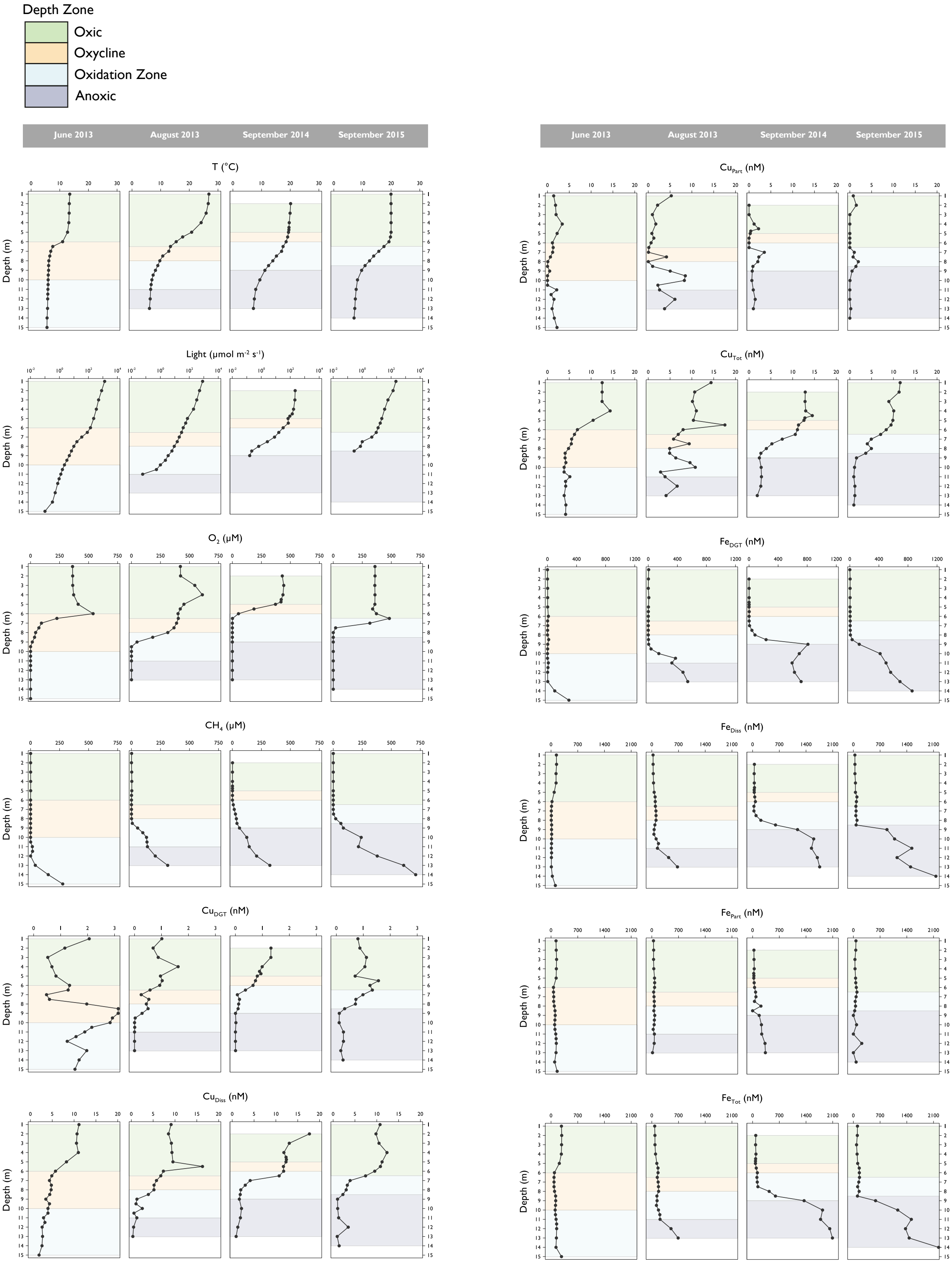

Depth Zone
Oxic
Oxycline
Oxidation Zone
Anoxic
June 2013
August 2013
September 2014
September 2015
T (°C)
Light (μmol m-2 s-1)
O2 (μM)
CH4 (μM)
CuDGT (nM)
CuDiss (nM)
CuPart (nM)
CuTot (nM)
FeDGT (nM)
FeDiss (nM)
FePart (nM)
FeTot (nM)
Depth (m)

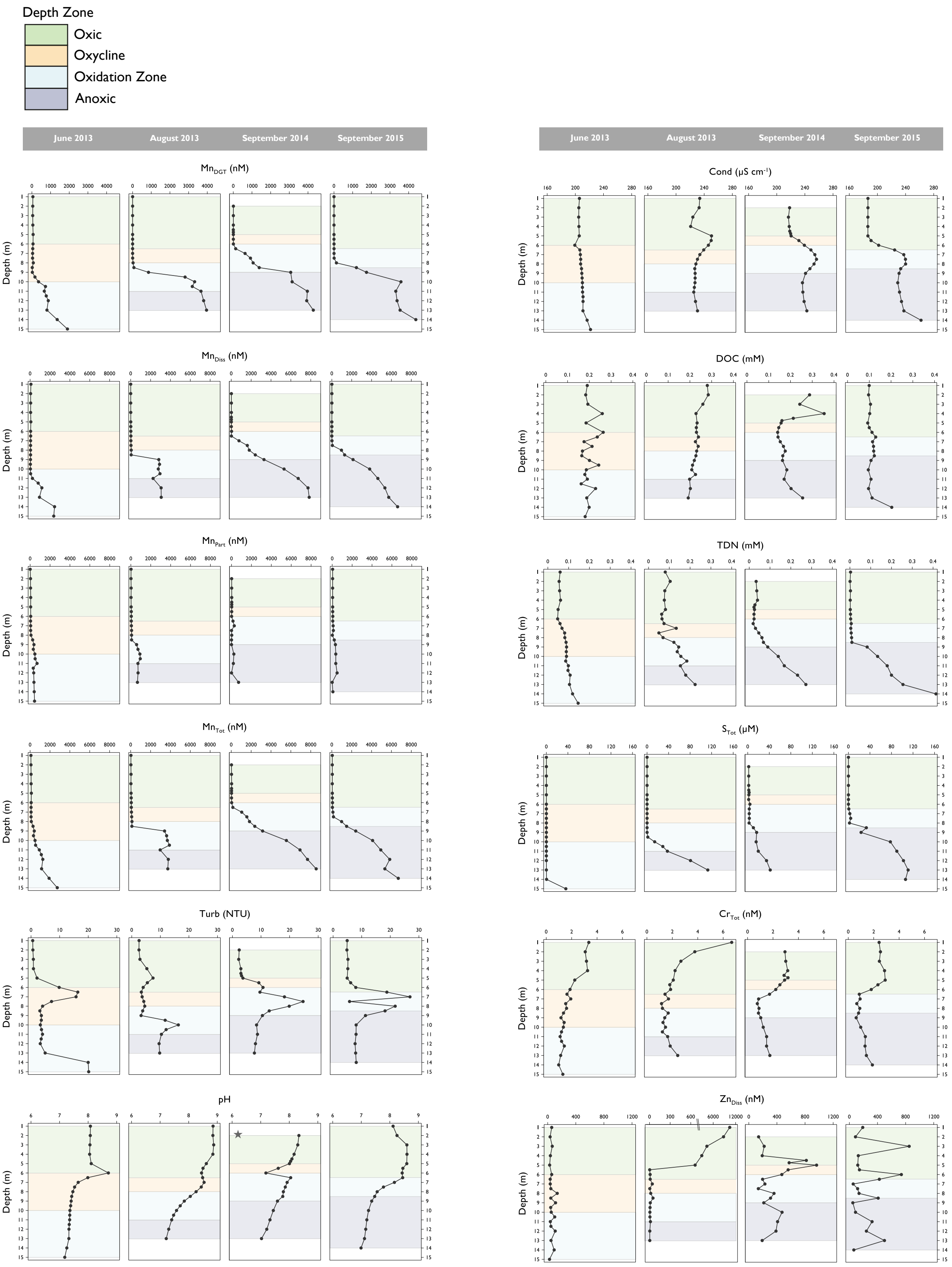
Depth Zone
Oxic
Oxycline
Oxidation Zone
Anoxic
June 2013
August 2013
September 2014
September 2015
Mn_DGT (nM)
Mn_Diss (nM)
Mn_Part (nM)
Mn_Tot (nM)
Turb (NTU)
pH
Cond (µS cm⁻¹)
DOC (mM)
TDN (mM)
S_Tot (µM)
Cr_Tot (nM)
Zn_Diss (nM)
Depth (m)

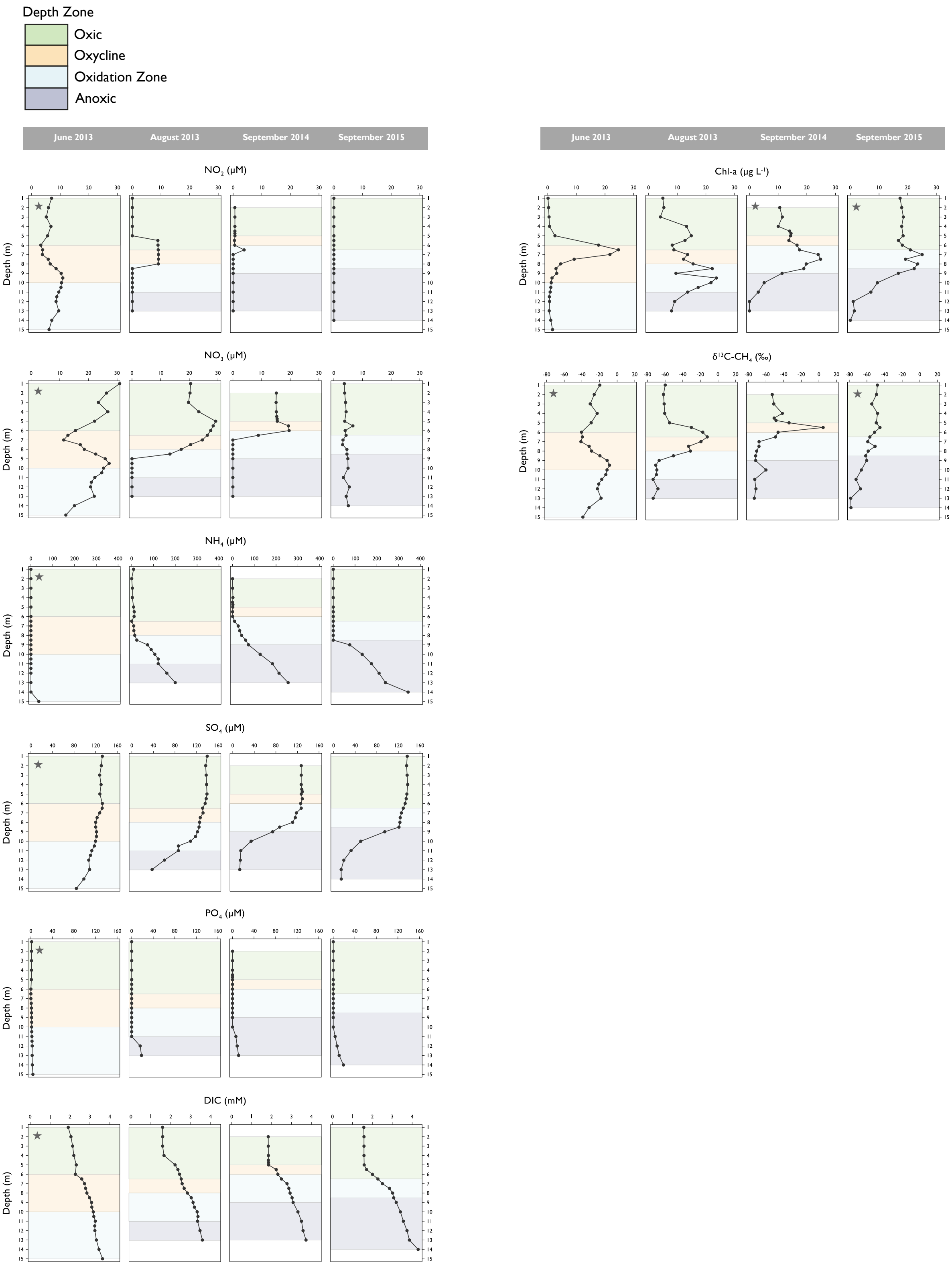

Depth Zone
Oxic
Oxycline
Oxidation Zone
Anoxic
June 2013
August 2013
September 2014
September 2015
NO2 (μM)
NO3 (μM)
NH4 (μM)
SO4 (μM)
PO4 (μM)
DIC (mM)
Chl-a (μg L-1)
δ13C-CH4 (‰)
Depth (m)

Figure S2

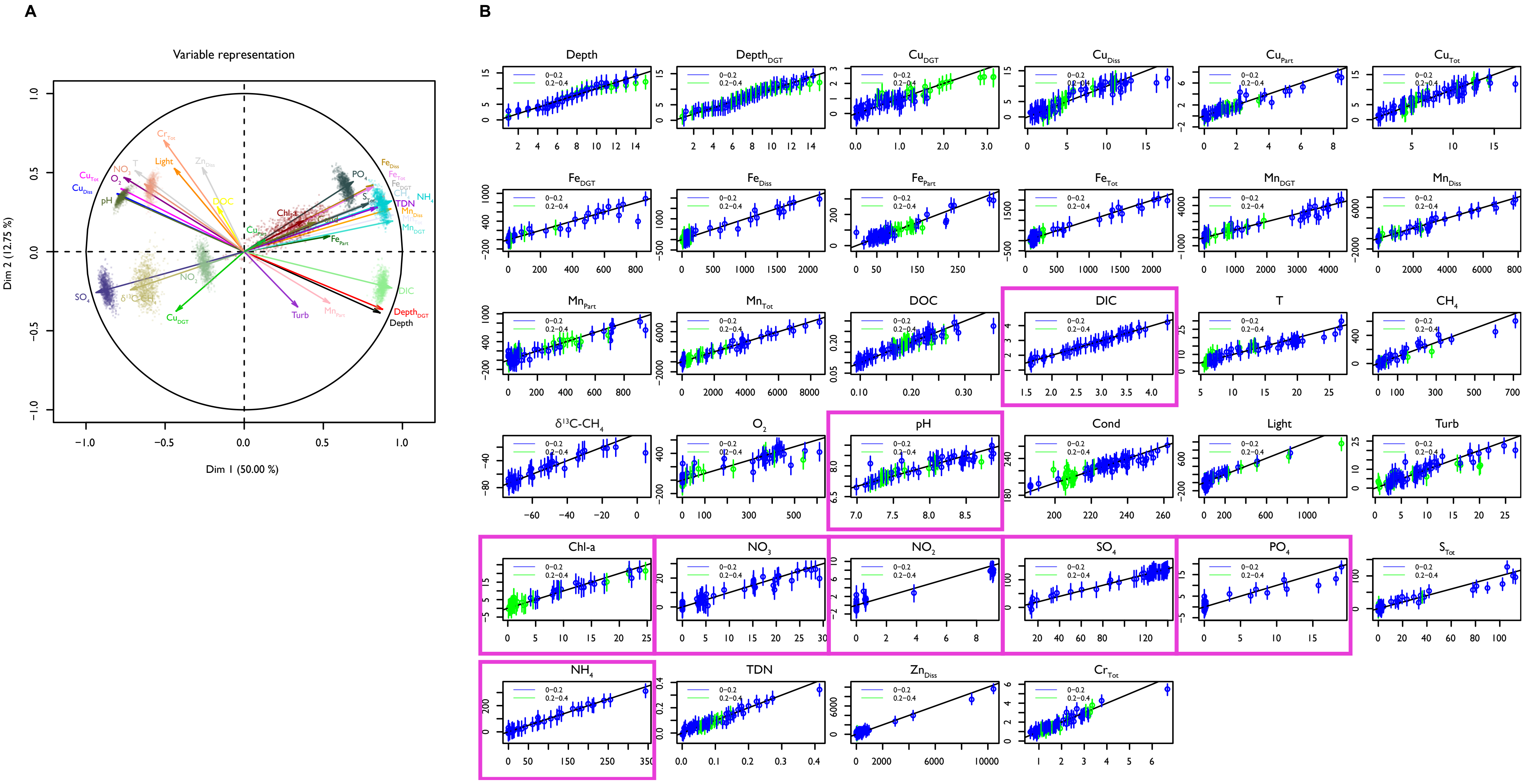
A
Variable representation
Dim 1 (50.00 %)
Dim 2 (12.75 %)
B
Depth
DepthDGT
CuDGT
CuDiss
CuPart
CuTot
FeDGT
FeDiss
FePart
FeTot
MnDGT
MnDiss
MnPart
MnTot
DOC
DIC
T
CH4
δ13C-CH4
O2
pH
Cond
Light
Turb
Chl-a
NO3
NO2
SO4
PO4
STot
NH4
TDN
ZnDiss
CrTot
0–0.2
0.2–0.4

Figure S3

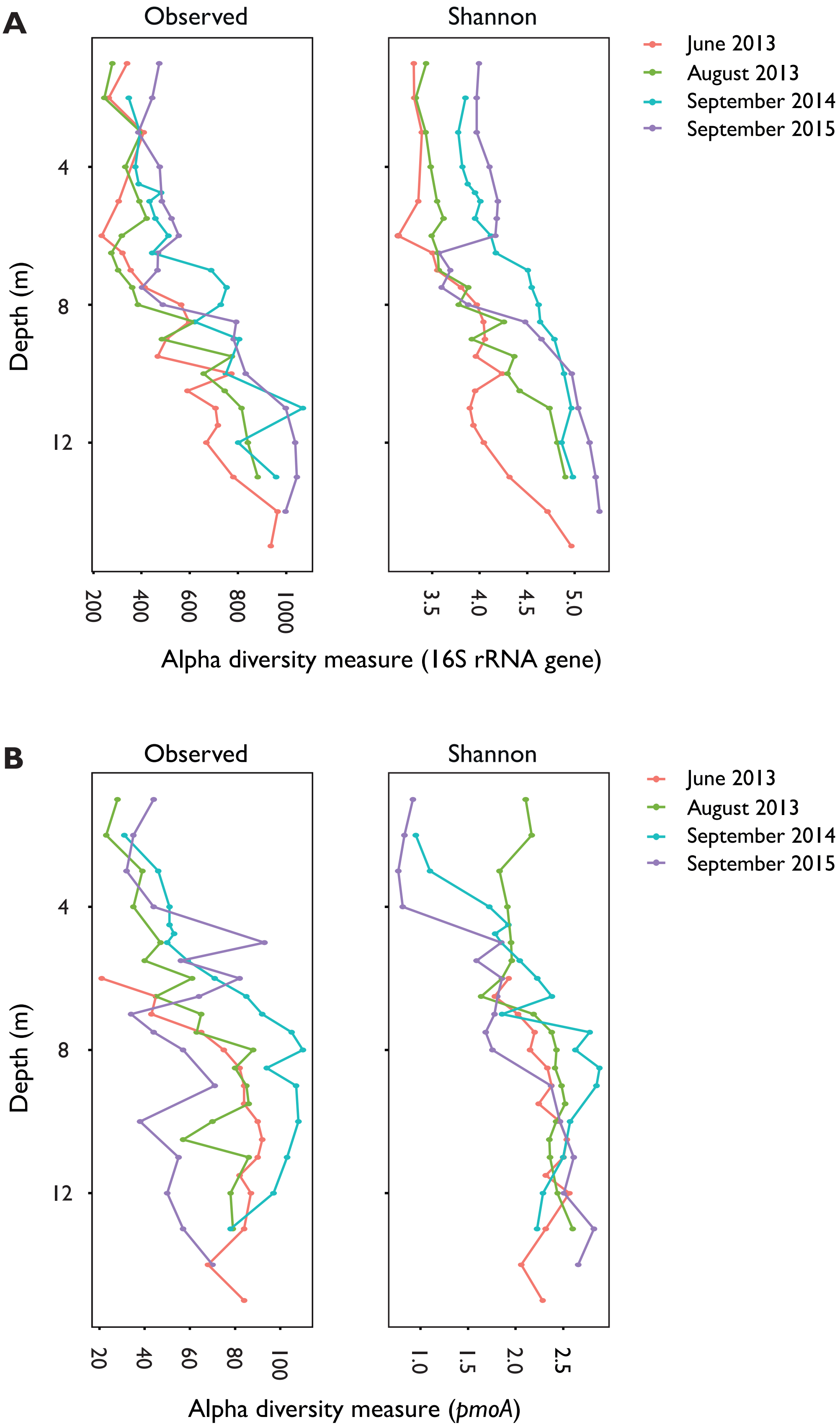
A
Observed
Shannon
June 2013
August 2013
September 2014
September 2015
Depth (m)
4
8
12
200
400
600
800
1000
3.5
4.0
4.5
5.0
Alpha diversity measure (16S rRNA gene)
B
Observed
Shannon
June 2013
August 2013
September 2014
September 2015
Depth (m)
4
8
12
20
40
60
80
100
1.0
1.5
2.0
2.5
Alpha diversity measure (pmoA)

Figure S4

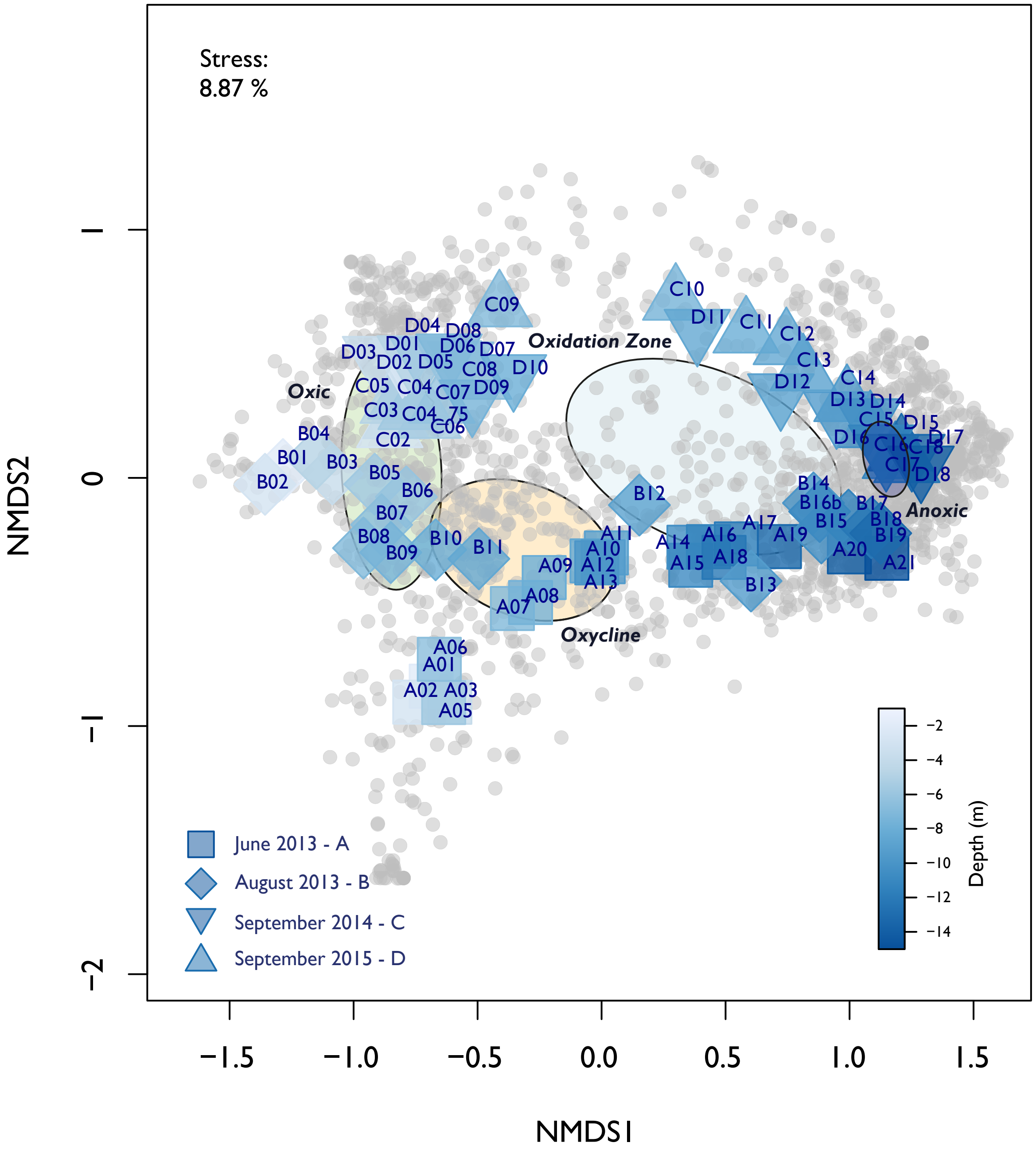

Stress:
8.87 %
Oxic
Oxidation Zone
Oxycline
Anoxic
NMDS1
NMDS2
June 2013 - A
August 2013 - B
September 2014 - C
September 2015 - D
Depth (m)

Figure S5

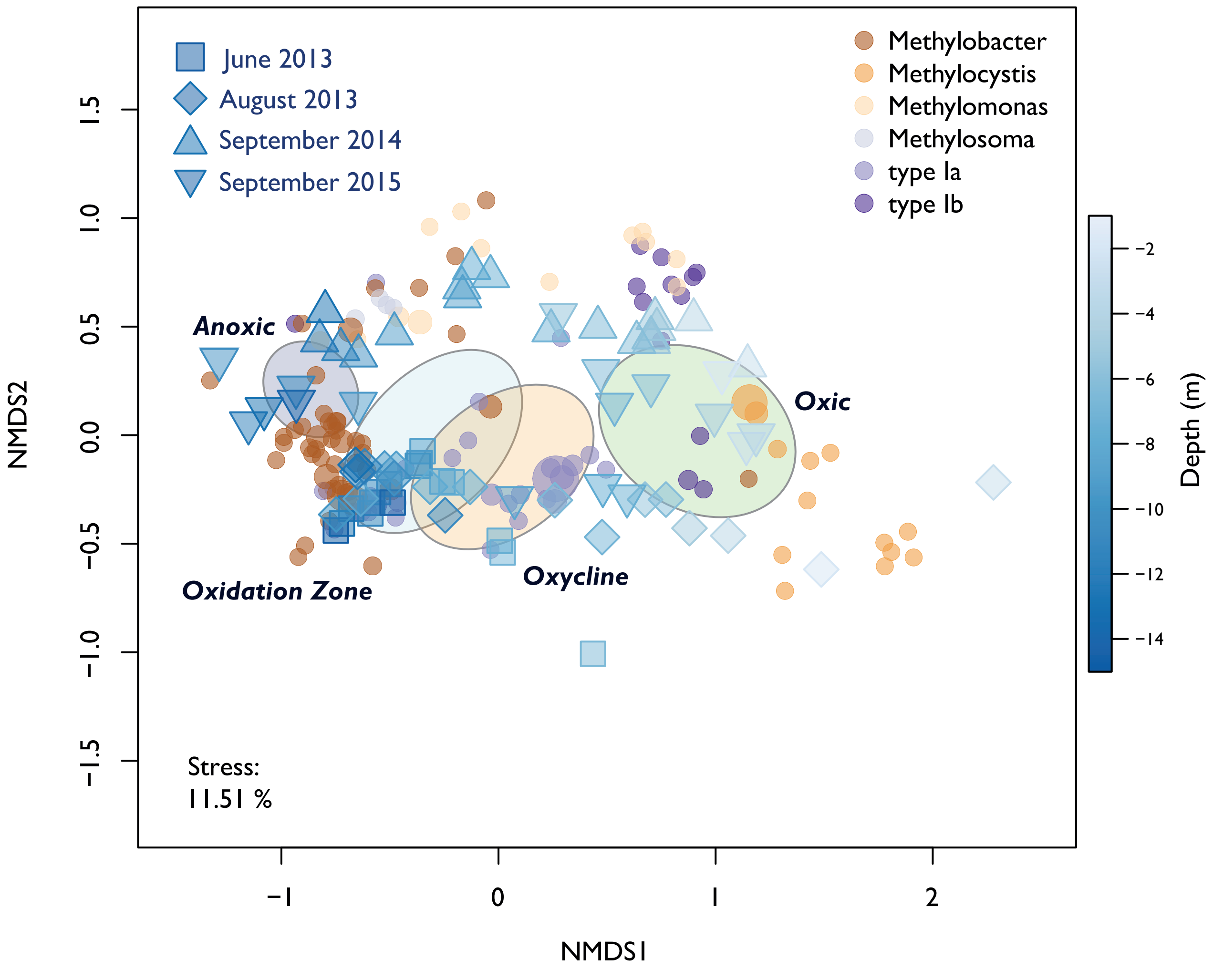
June 2013
August 2013
September 2014
September 2015
Methylobacter
Methylocystis
Methylomonas
Methylosoma
type Ia
type Ib
Anoxic
Oxic
Oxycline
Oxidation Zone
Stress:
11.51 %
NMDS1
NMDS2
Depth (m)
−1
0
1
2
1.5
1.0
0.5
0.0
−0.5
−1.0
−1.5
−2
−4
−6
−8
−10
−12
−14

Figure S6

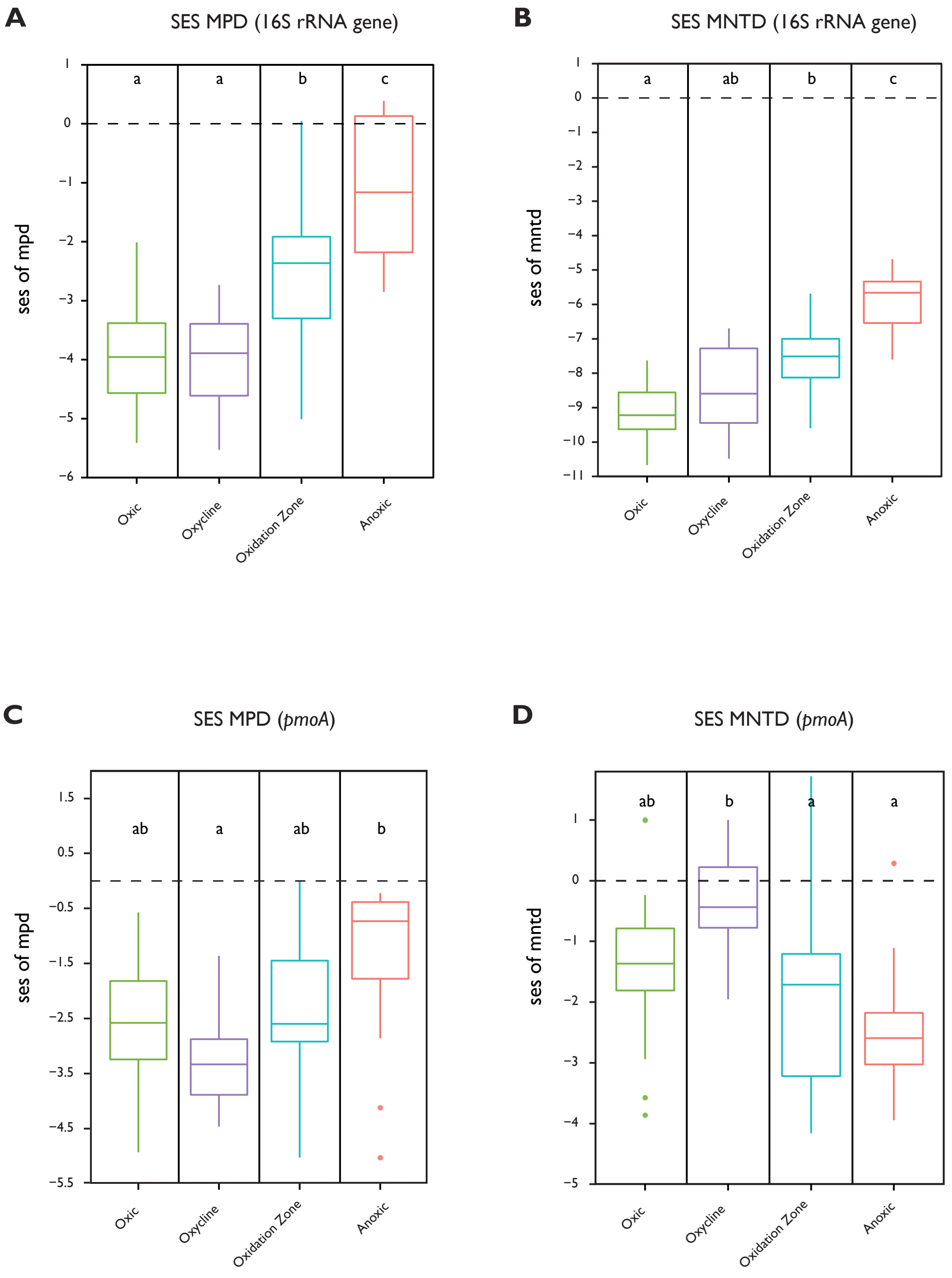
A
SES MPD (16S rRNA gene)
ses of mpd
a
a
b
c
Oxic
Oxycline
Oxidation Zone
Anoxic
B
SES MNTD (16S rRNA gene)
ses of mntd
a
ab
b
c
Oxic
Oxycline
Oxidation Zone
Anoxic
C
SES MPD (pmoA)
ses of mpd
ab
a
ab
b
Oxic
Oxycline
Oxidation Zone
Anoxic
D
SES MNTD (pmoA)
ses of mntd
ab
b
a
a
Oxic
Oxycline
Oxidation Zone
Anoxic
